# Low-Coordinate Iron Hydride Chemistry at an N,N,C-Heteroscorpionate Platform

**DOI:** 10.1021/acs.inorgchem.4c01596

**Published:** 2024-07-22

**Authors:** Addison Fraker, Brittany N. Linn, Alex McSkimming

**Affiliations:** † Department of Chemistry, Tulane University, New Orleans, Louisiana 70118, United States; ‡ Department of Chemistry, Massachusetts Institute of Technology, Cambridge, Massachusetts 02139, United States

## Abstract

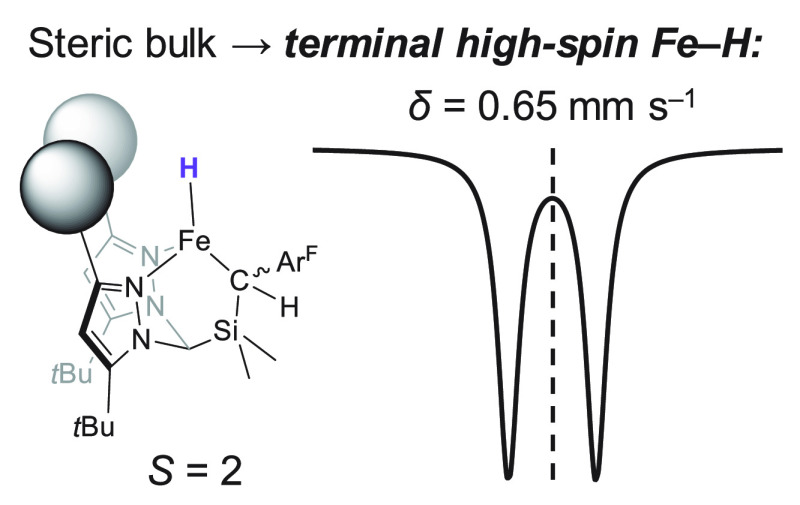

Locally high-spin iron hydrides are proposed to play a critical role as intermediates in iron–molybdenum cofactor (FeMoco)-catalyzed N_2_ fixation. Inspired by these biological systems, we report herein our initial investigations into low-coordinate iron hydride chemistry supported by our N,N,C-heteroscorpionate ligands. Those ligands with smaller steric profiles are unable to completely suppress the formation of a binuclear [Fe(μ_2_-H)]_2_ complex; however, the incorporation of more substantial steric bulk allows for the isolation of a rare example of a terminal, high-spin (*S* = 2) Fe^2+^ hydride. Fourier transform infrared spectroscopy suggests an unusually weak Fe–H bond in the latter molecule. Mössbauer spectroscopies, coupled with density functional theory calculations, highlights the substantial influence of the terminal hydride ligand on ^57^Fe isomer shift.

## Abstract 2

We report the synthesis and characterization of a rare example of a high-spin (*S* = 2) terminal iron hydride. Spectroscopic and computational analyses provide insight into the nature of the Fe−H bonding.

## Introduction

**Figure 1:**
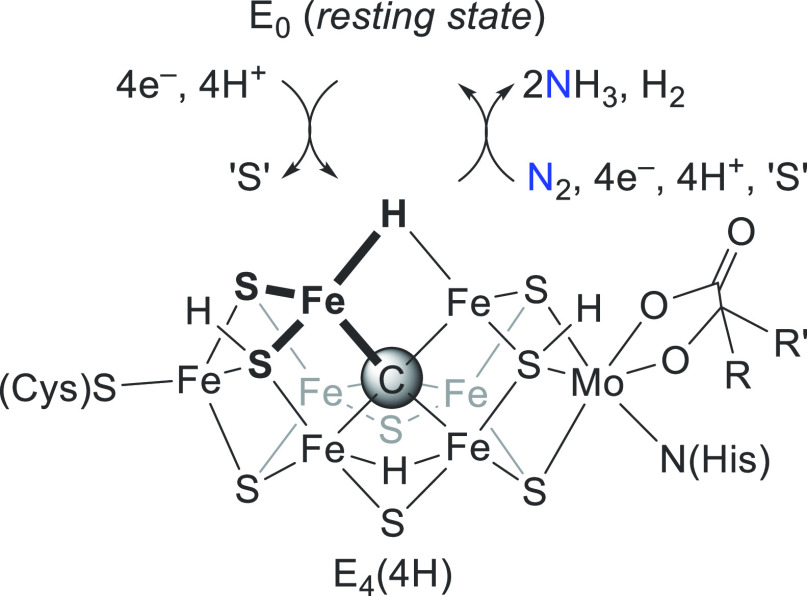
Reduction of N_2_ to NH_3_ at FeMoco via one (of many possible) E_4_(4H) states.

Hydride complexes of transition metals occupy a central position in synthetic inorganic chemistry.^1,2^ The overwhelming majority of such molecules are coordinately saturated and diamagnetic, often featuring 18-electron valence counts at the metal.^3,4^ The chemistry of the iron–molybdenum cofactor (FeMoco) of nitrogenase enzymes, which catalyzes reduction of N_2_ to NH_3_, is thus all the more conspicuous.^5^ Accumulation of four protons and electrons during turnover yields the so-called E_4_(4H) intermediate;^6^ this species contains two Fe–H sites that reductively eliminate H_2_ upon N_2_ binding the cofactor (Figure 1).^7^ Given that FeMoco is a relatively weak-field cluster, these hydride-bound Fe center(s) are expected to sample locally high spin state(s)—in stark contrast to the vast majority of synthetic metal hydrides.

As one might expect, in view of the intense and enduring study of FeMoco, a great deal of effort has been directed toward the preparation of low-coordinate (≤5), paramagnetic Fe hydrides.^8−27^ A recurring theme throughout this chemistry is the apparent preponderance of such species to adopt binuclear, [Fe(μ_2_-H)]_2_-type structures.^8,17,24^ As a result, only a handful of high-spin, terminal hydride complexes of Fe have been reported.^8,12,21^ Intermediate-spin, terminal Fe hydrides are similarly uncommon.^25−27^ The difficulties associated with the isolation of high-spin, terminal metal hydrides is by no means restricted to Fe: for instance, there are only two such Co hydrides,^28,29^ and no such examples for Mn. From a primarily fundamental perspective, therefore, our group has been drawn to this conspicuously limited class of molecules. More particularly, we are interested in the nature of the M–H bonding and its relationship to the electronic structure of the metal, which we deem to be a somewhat neglected aspect of these unusual species.

**Scheme 1:**
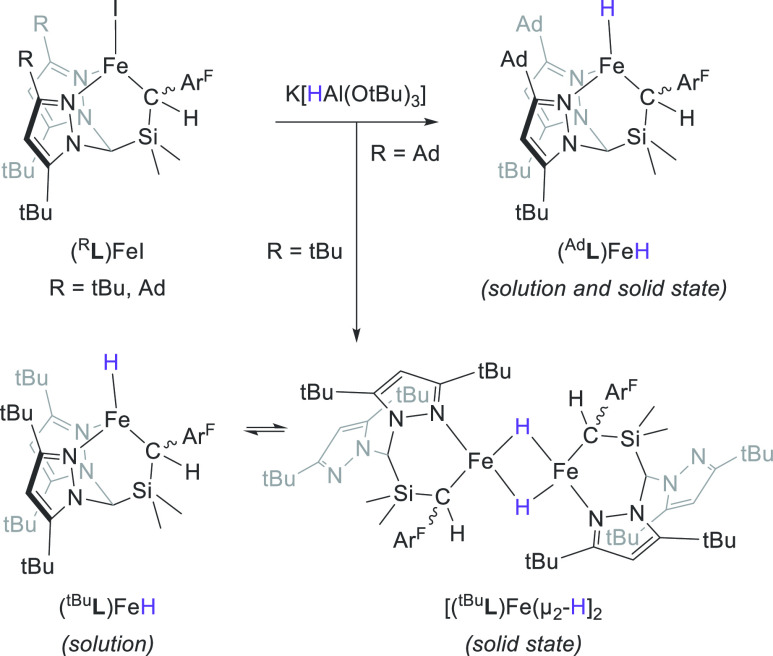
Synthesis of Fe Hydride Complexes Ar^F^ = 3,5-(CF_3_)_2_C_6_H_3_; Ad = 1-adamantyl.

Our lab has, over the past few years, developed a new class of N,N,C-heteroscorpionates (^R^**L**; where R denotes the metal-adjacent pyrazolyl substituents; Scheme 1). We naturally wondered if Fe–H complexes of these ligands could be prepared, and what the properties of these molecules might be. We thus report herein our initial forays into this chemistry: those ^R^**L** ligands with relatively small R substituents proved unable to entirely prevent formation of a binuclear [Fe(μ_2_-H)]_2_ complex; however, increasing the steric demands of these R groups gave rise to a rare example of a high-spin (*S* = 2), terminal Fe^2+^ hydride. Fourier transform infrared (FTIR) and Mössbauer spectroscopy, coupled with density functional theory (DFT) calculations, provide insight into the unusual terminal Fe–H interaction.

## Results and Discussion

We began our exploration into the Fe hydride chemistry of the ^R^**L** ligand system by reacting (^tBu^**L**)FeCl^30^ with a variety of reagents reported to generate 3d metal hydrides from the corresponding chlorides, such as [HBEt_3_]^−^, [HB(sBu)_3_]^−^, and Na[H_2_Al(OCH_2_CH_2_OCH_3_)_2_]. In all cases only mixtures of diamagnetic species were obtained. We have found, over the course of other synthetic work, that replacing Cl with I in (^R^**L**)MX precursors greatly improves the outcome of reductive chemistry.^31^ Thus, (^tBu^**L**)FeI was prepared from (^tBu^**L**)Li and FeI_2_THF_2.67_^32^ as a yellow crystalline solid in 59% yield. This complex was fully characterized and, as expected, is structurally and spectroscopically much the same as (^tBu^**L**)FeCl. The reactivity of (^tBu^**L**)FeI, however, transpired to be markedly different: exposure to K[HB(sBu)_3_] did indeed generate a new paramagnetic species (by ^19^F NMR spectroscopy), in addition to a large amount of decomposition product(s). It is worth noting that [HBEt_3_]^−^ sources gave no detectable amount of any paramagnetic material, despite often being the “go-to” hydride reagent for inorganic chemists. Serendipitously, we managed to obtain a small quantity of poor quality, dark orange crystals from the reaction of (^tBu^**L**)FeI with K[HB(sBu)_3_], which allowed us to identify the product of this reaction as the binuclear, bridging dihydride complex [(^tBu^**L**)Fe(μ_2_-H)]_2_ (Figure 1; good quality crystals were obtained after we formulated a more optimized preparation, see below). The Fe sites in this molecule are rendered equivalent as a result of a crystallographic inversion center. The bridging binding mode for the hydrides is accommodated through decoordination of a single pyrazole donor; thus, each Fe site remains four coordinate and roughly tetrahedral (τ_4_ = 0.91).^33^ This structure again highlights how a hemilabile pyrazole donor in ^R^**L** heteroscorpionates can facilitate large changes in bonding at the unique ligand site.^30^ The hydrido ligands were located in the difference map and appear slightly asymmetrical in their coordination to each Fe site (*d*(Fe–H) = 1.73(2), 1.75(2) Å); owing to poor precision in the Fe–H distances, however, this cannot be stated with certainty. Nevertheless, we note that this structural feature, as well as the approximate geometry at the Fe sites, is in good agreement with broken-symmetry (BS) DFT calculations performed on the truncated model complex [(*L)Fe(μ_2_-H)]_2_ (calcd. *d*(Fe–H) = 1.75, 1.80 Å; τ_4_ = 0.92; where *L has all 3,5-pyrazole groups replaced by −CH_3_; see the Experimental Section for details). Although utmost care should be made in any interpretation of M–H distances gleaned from XRD measurements, these values are also roughly in line with those determined for other electronically and geometrically similar, four-coordinate [Fe(μ_2_-H)]_2_ complexes.^16,17,24^ Magnetic susceptibility measurements on solid samples of [(^tBu^**L**)Fe(μ_2_-H)]_2_ at 295 K provide a magnetic moment of 5.0 ± 0.2 μ_B_, well below the expected spin-only moment for two magnetically isolated or ferromagnetically coupled high-spin Fe^2+^ centers. This suggests relatively weak antiferromagnetic exchange between the Fe sites, as reported for analogous species,^16,17,24^ and is in line with our calculations (see the Experimental Section).

As the above reaction of (^tBu^**L**)FeI with K[HB(sBu)_3_] was so poor yielding as to be impractical for preparative purposes, we set about exploring a means by which [(^tBu^**L**)Fe(μ_2_-H)]_2_ could be generated on scale. Ultimately, we discovered that, although commercially available Li[HAl(OtBu)_3_] did not react with (^tBu^**L**)FeI, the potassium salt of this reagent—K[HAl(OtBu)_3_]^34^—did, and cleanly, presumably due to the additional driving force imparted by KI precipitation (Scheme 1). It is worth emphasizing the quite remarkable synthetic utility of this neglected reagent for this reaction; going forward, we expect K[HAl(OtBu)_3_] to find broad use in preparing other 3d metal hydrides.

During characterization of [(^tBu^**L**)Fe(μ_2_-H)]_2_ we were initially frustrated by the presence of NMR resonances for what appeared to be a very minor, ostensibly weakly paramagnetic, impurity. This substance could not be removed even through multiple, careful recrystallizations from a variety of different solvents. We also noted that solutions of [(^tBu^**L**)Fe(μ_2_-H)]_2_ gave rise to ^1^H NMR signals that were very similar in width and chemical shift to those observed for (^tBu^**L**)FeI, as well as very similar magnetic moments (∼5.2 μ_B_ per Fe). We consequently surmised that, in solution, [(^tBu^**L**)Fe(μ_2_-H)]_2_ dissociates almost entirely to afford the terminal, high-spin hydride complex (^tBu^**L**)FeH. Peaks for the “impurity” we invariably observed in solution samples of [(^tBu^**L**)Fe(μ_2_-H)]_2_ were seen to ablate upon dilution (Figure S17), consistent with these being due to a small amount of persistent [(^tBu^**L**)Fe(μ_2_-H)]_2_. Such a phenomenon has been reported for [(NacNac)Fe(μ_2_-H]_2_ (NacNac = β-diketiminate), which likewise can exist as an equilibrium mixture of both dimeric and monomeric forms in solution with the monomer favored at low concentrations and high temperatures.^8^ Given ^1^H NMR resonances for both [(^tBu^**L**)Fe(μ_2_-H)]_2_ and (^tBu^**L**)FeH are observed in solution simultaneously, conversion between the two must be relatively slow on the NMR experiment time scale. This is unsurprising, given the large reorganization energy very likely associated with breaking apart the two (^tBu^**L**)FeH units and subsequent recoordination of the pyrazole donors. The poor thermal stability of [(^tBu^**L**)Fe(μ_2_-H)]_2_ precluded NMR experiments at high temperatures, and at accessible low temperatures the concentration of [(^tBu^**L**)Fe(μ_2_-H)]_2_ changes only marginally. In addition, the ratio of monomer to dimer is essentially independent of solvent; e.g. C_6_H_6_ vs THF. As a result, we have been unable to obtain any quantitative thermodynamic data regarding the [(^tBu^**L**)Fe(μ_2_-H)]_2_ ⇌ (^tBu^**L**)FeH equilibrium.

**Figure 2:**
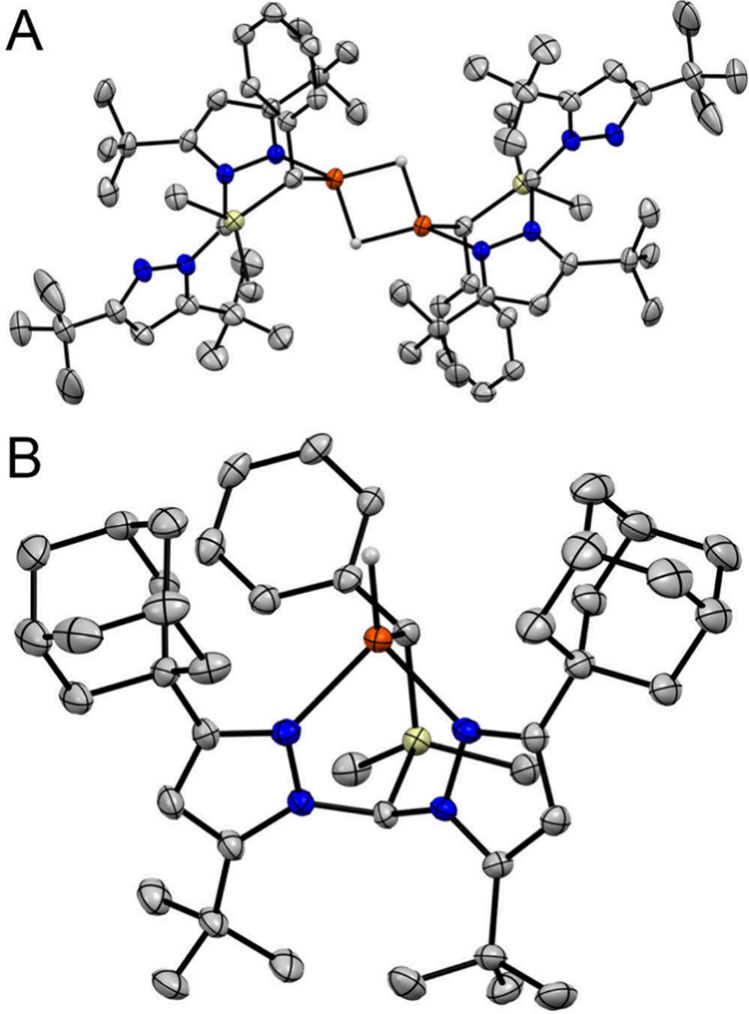
Thermal ellipsoid plots (50%) of (A) [(^tBu^**L**)Fe(μ_2_-H)]_2_ and (B) (^Ad^**L**)FeH. Orange, blue, yellow, and gray ellipsoids represent Fe, N, Si, and C, respectively. Hydrogen atoms except those bound to Fe, solvent molecules, and CF_3_ groups are omitted for clarity.

In light of the above observations, we were obviously keen to explore the possibility of preparing an exclusively mononuclear high-spin Fe hydride complex, (^R^**L**)FeH. Inspection of the structure of [(^tBu^**L**)Fe(μ_2_-H)]_2_ suggested to us that increasing the size of the pyrazole R substituents would act to suppress formation of such binuclear species. Consequently, using methodologies well-established by our lab, we prepared the considerably more hindered ligand precursor, ^Ad^**L**H, in which the metal-adjacent tBu groups are replaced by larger 1-adamantyl substituents (see the Experimental Section). Metalation of ^Ad^**L**H proceeds smoothly to afford (^Ad^**L**)FeI (Scheme 1), which, as expected, does not differ substantially from (^tBu^**L**)FeI. Gratifyingly, reaction of (^Ad^**L**)FeI with excess K[HAl(OtBu)_3_] gave the bright yellow, terminal hydride complex (^Ad^**L**)FeH in good yield (72%; Scheme 1 and Figure 2). The solution state magnetic moment of (^Ad^**L**)FeH (5.3 μ_B_) is similar to that obtained for (^Ad^**L**)FeI and is consistent with a high-spin state (*S* = 2) at Fe. Concordantly, calculations performed on (*L)FeH suggest the intermediate spin state (*S* = 1) to be substantially higher in energy (by ∼14 kcal mol^–1^; see the Experimental Section), and with the Fe distorted toward a more square planar arrangement (τ_4_ = 0.42). X-ray diffraction (XRD) analysis at 150 K reveals the Fe–donor distances and Fe geometry to change only marginally upon exchanging −Cl/I for −H (Table 1),^30^ indicating (^Ad^**L**)FeH remains high-spin down to at least 150 K. Mössbauer spectra obtained for (^Ad^**L**)FeH show only a small, expected temperature dependence^35^ from 150 to 5 K (Figure S31), without the emergence of any new resonance(s), thus ruling out spin-crossover behavior.^36^

**Table 1:** Selected Experimental and Calculated Parameters for (^tBu^**L**)FeI, (^Ad^**L**)FeH, and [(^tBu^**L**)Fe(μ_2_-H)]_2_^a^

	(^tBu^**L**)FeI	(^Ad^**L**)FeI	(^Ad^**L**)FeH	[(^tBu^**L**)Fe(μ_2_-H)]_2_
Fe–C_alkyl_ (Å)	2.097(2)	2.108(3)	2.121(3)	2.076(2)
Fe–N_pz1_ (Å)	2.133(2)	2.148(2)	2.149(2)	2.090(2)
Fe–N_pz2_ (Å)	2.120(2)	2.110(1)	2.116(3)	
δ (mm s^–1^)	0.81 (0.72^a^, 0.75^b^, 0.71^c^)		0.65 (0.49^a^, 0.54^b^, 0.52^c^)	0.53 (0.42^a^, 0.48^b^, 0.46^c^)
|Δ*E*_Q_| (mm s^–1^)	2.14 (1.84^a^, 1.98^b^, 2.13^c^)		1.08 (1.88^a^, 1.76^b^, 1.79^c^)	3.18 (2.37^a^, 2.45^b^, 2.83^c^)

a For Mössbauer parameters, the numbers in parentheses are calculated; see the Experimental Section for details: ^a^BP86; ^b^TPSSh; ^c^B3LYP.

(^Ad^**L**)FeH is distinct from other reported terminal high-spin Fe^2+^ hydrides (and (^tBu^**L**)FeH), being homogeneous in both the solid state and in solution, with the hydrido ligand entirely free of secondary contacts.^8,21^ As for [(^tBu^**L**)Fe(μ_2_-H)]_2_, the hydrido ligand was located in the electron density difference map a distance of 1.72(2) Å from the Fe center. This would imply a somewhat longer bond than that predicted by DFT (calcd. *d*(Fe–H) = 1.64 Å), highlighting the need for caution in extrapolating actual M–H bond lengths from XRD studies. The Fe center in (^Ad^**L**)FeH is roughly tetrahedral (τ_4_ = 0.81), with the hydrido ligand tilted slightly away from the C_alkyl_ donor. Allowing the position of the hydride to freely relax along the ground-state potential energy surface in calculations performed on (*L)FeH reasonably well-reproduces the crystallographically determined N_pz_/C_alkyl_–Fe–H angles; in particular, the relatively obtuse ∠C_alkyl_–Fe–H (expt. = 124.8(8)°; calcd. = 130°).

The presence of the Fe–H moiety in (^Ad^**L**)FeH was unequivocally determined by FTIR spectroscopy, which shows a broad, somewhat weak resonance for the Fe–H stretch at 1576 cm^–1^ (Figure S29). This band is absent for (^Ad^**L**)FeD; however, the corresponding Fe–D stretch is unfortunately obscured by an intense peak that occurs at roughly the same expected frequency (i.e., ∼1114 cm^–1^). The DFT calculated value of 1633 cm^–1^ for ν(Fe–H) is in reasonable agreement with experiment, although does suggest a slight overestimation of Fe–H covalency. To the best of our knowledge, this represents the first unambiguous IR characterization of a hydride terminally bound to high-spin Fe. The Fe–H stretching frequency for (^Ad^**L**)FeH is, predictably, considerably lower than observed for low-spin Fe hydrides (ν(Fe–H) > 1850 cm^–1^).^4,37^ It is also appreciably lower than seen in square planar, intermediate-spin terminal Fe hydrides;^25−27^ e.g. for (PNP)FeH, ν(Fe–H) = 1671 cm^–1^ (PNP = a bis(phosphino)pyrrolide pincer ligand).^25^ This suggests the Fe–H bond in (^Ad^**L**)FeH to be unusually weak and, potentially, labile; the reactivity of this molecule will be reported in due course. In contrast to the terminal hydride complex, FTIR spectra for [(^tBu^**L**)Fe(μ_2_-H)]_2_ and [(^tBu^**L**)Fe(μ_2_-D)]_2_ are essentially identical. The Fe–H/D stretching bands in these binuclear species are expected to be far less intense,^37^ which has been similarly documented for analogous [(NacNac)Fe(μ_2_-H/D)]_2_ complexes.^38^ Finally, we briefly note that we have, thus far, been unable to detect a resonance for any hydride/deuteride ligand by ^1^H/^2^H NMR spectroscopy.

**Figure 3:**
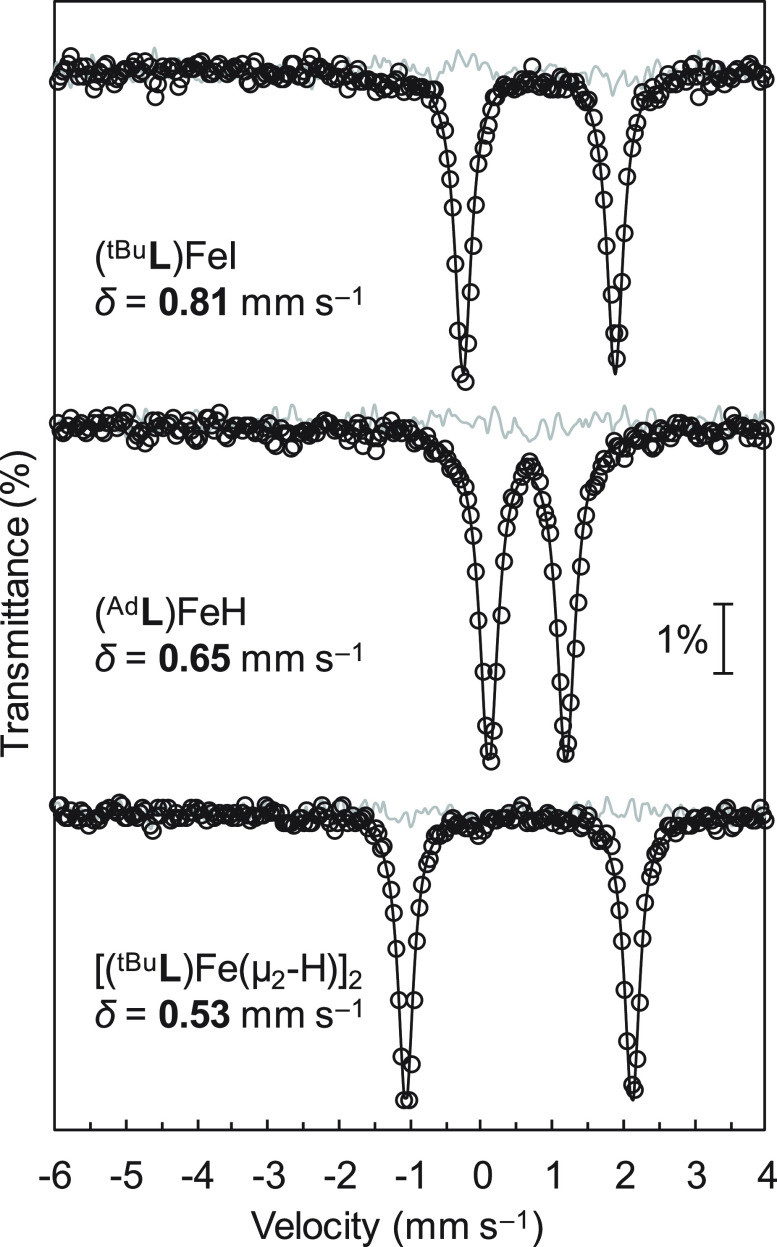
Zero-field ^57^Fe Mössbauer spectra (80 K) collected for the shown complexes. Black circles = experimental data; solid trace = simulations; gray trace = residuals. For simulation parameters, see Table 1.

To further our understanding of the electronic structure of our complexes, ^57^Fe Mössbauer spectra were collected (Figure 3); experimental and calculated data, as well as Fe–^R^**L** bond lengths from XRD studies are summarized in Table 1. Before presenting our results, we provide some brief background. Mössbauer isomer shifts (δ) are linearly related to the ground state electron density at the Fe nucleus (ρ_0_), which can be broken down into core (ρ_0_^1s^ + ρ_0_^2s^ + ρ_0_^3s^) and valence (ρ_0_^val^) contributions, with the latter arising due to mixing of the otherwise unoccupied Fe(4s) orbital with ligand-based molecular orbitals.^39^ The Fe(1s) and Fe(2s) electrons contribute the vast majority (≈99%) of the electron density, and are roughly consistent over most Fe complexes. Contributions from ρ_0_^3s^ and ρ_0_^val^ constitute the remaining 1%, and these two values—particularly ρ_0_^val^—account for the majority of variations in isomer shifts. Increases in ρ_0_^val^ correlate well with changes in the shape of valence orbitals with 4s character, which, in turn, is caused by enhanced Fe-ligand covalency and shorter Fe–ligand bonds.^39^

For the mononuclear complexes, replacing iodide with hydride causes an appreciable decrease in Mössbauer isomer shift: 0.81 mm s^–1^ for (^tBu^**L**)FeI: cf. 0.65 mm s^–1^ for (^Ad^**L**)FeH. As the Fe–N_pz_ and Fe–C_alkyl_ distances differ only slightly between these two complexes, we attribute this to the markedly shorter Fe–H vs Fe–I bond.^40^ The Mössbauer spectrum obtained for solid [(^tBu^**L**)Fe(μ_2_-H)]_2_ reveals a single quadrupole doublet with an isomer shift of 0.53 mm s^–1^, distinctly lower than that observed for (^Ad^**L**)FeH. So, too, do the quadrupole splittings for these complexes differ markedly: 1.08 mm s^–1^ for (^Ad^**L**)FeH cf. 3.18 mm s^–1^ for [(^tBu^**L**)Fe(μ_2_-H)]_2_. Evidently, then, [(^tBu^**L**)Fe(μ_2_-H)]_2_ is exclusively binuclear in the solid-state, despite preferring to dissociate in solution to give (^tBu^**L**)FeH. The isomer shift for [(^tBu^**L**)Fe(μ_2_-H)]_2_ is appreciably lower than that observed for the analogous complex [(NacNac)Fe(μ_2_-H)]_2_ (δ = 0.66 mm s^–1^),^16^ which is presumably a result of the more covalent Fe–^R^**L** vs Fe–NacNac interactions.

DFT calculations are in qualitative agreement with the experimental Mössbauer data, and reproduce the observed parameters reasonably well. Although a number of calibrations are available for the conversion of DFT computed ρ_0_ values into Mössbauer isomer shifts,^41,42^ we have opted to use that given by Neese,^43^ which we perceive to have the least bias in the training set. We have examined the performance of several common functionals (BP86, TPSSh, and B3LYP), with TPPSh providing the best predictive power; thus, we have utilized this functional throughout this work. For each of the functionals, the calculated isomer shifts are consistently lower than observed experimentally, with those provided by the pure functional BP86 (i.e., 0% Hartree–Fock exchange) the lowest. This is unsurprising, given pure functionals tend to overestimate metal–ligand covalency,^44^ and this is clearly evidenced by the greater relative contributions of ρ_0_^val^ to ρ_0_ for BP86 cf. TPSSh and B3LYP (Table S1). Although our current calculations seem to suggest DFT has a general tendency to overestimate Fe–ligand covalency in these molecules, a more concrete assessment will require analysis of Mössbauer data obtained for additional (^R^**L**)Fe complexes.

Although ρ_0_^val^ provides an overall picture of Fe-ligand covalency, considering localized molecular orbitals for a particular Fe complex allows this term to be analyzed in terms of the individual contributions from each Fe–donor interaction.^45^ We were curious, then, to ascertain the specific impact of the hydride ligands on the isomer shifts calculated for [(*L)Fe(μ_2_-H)]_2_ and (*L)FeH.

Table 2 provides a breakdown of ρ_0_ for our two model hydride complexes, including a further breakdown of ρ_0_^val^ into contributions from each Fe–L bond. As expected, these do indeed reveal a greater value of ρ_0_^val^ for [(*L)Fe(μ_2_-H)]_2_ cf. (*L)FeH. This accounts for only the minor part of the overall difference in ρ_0_ for these two complexes, with increases in ρ_0_^2s^ + ρ_0_^3s^—and to a lesser extent, ρ_0_^1s^—giving rise to the remainder. This would typically be ascribed to a deshielding mechanism, whereby decreased population of the Fe(3d) orbitals less effectively screens the core Fe(s) orbitals from nuclear charge.^39^ The Fe(3d) Löwdin population for [(*L)Fe(μ_2_-H)]_2_ is calculated, however, to be greater than that for (*L)FeH: 6.96 vs 6.81 e^–^, respectively. This apparent anomaly suggests that any shielding imparted by the more filled Fe(3d) shell in [(*L)Fe(μ_2_-H)]_2_ is counteracted by Fe(s) contraction induced by the overall shorter Fe–ligand interactions.

**Table 2:** Calculated Contributions (TPSSh) to the Core Fe Electron Density (ρ_0_) for (*L)FeH and [(*L)Fe(μ_2_-H)]_2_^a^

	(*L)FeH	[(*L)Fe(μ_2_-H)]_2_
ρ_0_^1s^	10475.9826	10475.9911
ρ_0_^2s^	968.9110	968.9429
ρ_0_^3s^	133.7549	133.8029
ρ_0_(Fe–N_pz1_)^a^	0.4110	0.5420
ρ_0_(Fe–N_pz2_)^a^	0.3921	-
ρ_0_(Fe–C_alkyl_)^a^	1.2219	1.4637
ρ_0_^val^(σ(Fe–^R^**L**))	2.0250	2.0057
ρ_0_(Fe–H^1^)^a^	2.0079	1.0791
ρ_0_(Fe–H^2^)^a^	-	0.8382
ρ_0_^val^(σ(Fe–L))	4.0329	3.9230
ρ_0_^val^(σ(Fe–Fe))	-	0.1581
ρ_0_^val^(other)^b^	0.2158	0.2410
ρ_0_^val^	4.2487	4.3221
ρ_0_	11582.8972	11583.0590

a Values are given in au: ^a^summed over α and β manifolds; ^b^sum of contributions, none of which is >0.04 au.

We predicted, a priori, the greater value of ρ_0_^val^ for [(*L)Fe(μ_2_-H)]_2_ would be a result of (i) the modestly shortened Fe–C_alkyl_ and Fe–N_pz_ bonds cf. (*L)FeH (Δ*d*(Fe–C_alkyl_) = −0.045(3) Å; av. Δ*d*(Fe–N_pz_) = −0.043(4) Å) and (ii) a relatively ionic Fe–N_pz_ bond in (*L)FeH being replaced by a more covalent Fe–(μ_2_-H) interaction in [(*L)Fe(μ_2_-H)]_2_. For the binuclear complex [(*L)Fe(μ_2_-H)], the shorter Fe–N_pz_ and Fe–C_alkyl_ bonds indeed contribute more to ρ_0_^val^ than do the analogous interactions in (*L)FeH. This, however, is offset almost exactly by the presence of the additional pyrazole donor in the mononuclear complex. Interestingly, the contribution to ρ_0_^val^ of the terminal hydrido ligand in (*L)FeH is roughly twice that of a single bridging hydride in [(*L)Fe(μ_2_-H)]_2_. When taken together, the two bridging hydrides contribute much the same to ρ_0_^val^ as the single terminal hydride. We were somewhat surprised by the dramatic impact the terminal hydrido ligand is calculated to have on ρ_0_^val^ and, by extension, the Mössbauer spectrum of (^Ad^**L**)FeH. Indeed, the terminal hydride is calculated to contribute a factor of ∼1.6 more to ρ_0_^val^ than the likewise strongly σ-basic alkyl donor. Seeing as DFT predictions for the isomer shift of (^Ad^**L**)FeH are less accurate than those for [(^tBu^**L**)Fe(μ_2_-H)]_2_, and in light of our FTIR data (see above), it seems possible that Fe–H bonding in the former is less covalent than DFT might suggest.

As a whole, then, and perhaps surprisingly, the Fe–donor interactions in [(*L)Fe(μ_2_-H)]_2_ contribute slightly less to ρ_0_^val^ than they do for (*L)FeH. Rather, the greater ρ_0_^val^ observed for [(*L)Fe(μ_2_-H)]_2_ stems primarily from a set of poorly overlapping σ(Fe(3d)–Fe(3d)) orbitals. We interpret these contributions to be a result of the calculated antiferromagnetic exchange, which can be regarded as, effectively, weak Fe–Fe bonding. Although having a relatively subtle, if decisive influence here, we expect such contributions to ρ_0_^val^ to play an important role in determining Mössbauer isomer shifts for more strongly coupled hydride-bridged Fe complexes.

## Conclusions

The strongly σ-donating heteroscorpionates developed by our group have, subject to the degree of steric encumbrance, proven capable of supporting both binuclear and mononuclear four-coordinate Fe hydrides. The latter molecule, (^Ad^**L**)FeH, is particularly noteworthy, being a rare example of a terminal hydride complex of high-spin (*S* = 2) Fe. Successful formation of our hydride complexes from the corresponding halide required: a) use of iodide, as opposed to chloride, as the leaving group; and b) careful screening of a number of hydride reagents and reaction solvents, most of which lead to no product formation at all. Our work here thus serves to emphasize the nuances associated with this branch of inorganic syntheses, and introduces K[HAl(OtBu)_3_] as a potentially useful reagent for others exploring similar chemistry. FTIR spectroscopy reveals the Fe–H bond in (^Ad^**L**)FeH to be unusually weak compared to diamagnetic and intermediate-spin terminal Fe hydrides, which we anticipate will lead to a rich reaction chemistry for this molecule. Mössbauer data, in concert with DFT, suggests the terminal Fe–H interaction in (^Ad^**L**)FeH to impart a substantial impact on ^57^Fe isomer shift, but perhaps less so than the calculations would indicate. Future work will aim to further our understanding of Fe–H bonding in this, and other, high-spin Fe hydrides.

## Experimental Section

### General Methods

All manipulations involving metal complexes were carried out in an N_2_ atmosphere glovebox. Glassware was oven-dried for at least several hours at 160 °C prior to use.

### Materials

All solvents except *n*-pentane and 1,2-difluorobenzene (DFB) were distilled from purple Na/benzophenone prior to use. All solvents were stored over activated 3 Å molecular sieves for at least 24 h prior to use. All reagents were purchased from commercial suppliers and used without further purification unless otherwise noted. ^tBu^**L**H^30^ was prepared according to literature procedures. An alternative synthesis of K[H/DAl(OtBu)_3_] that does not require K[AlH_4_]^34^ that we have found to be most convenient is presented below, with spectroscopic data given in the Supporting Information (SI).

### Comment on Purity

Organic products were deemed pure by ^1^H NMR spectroscopy. All metal complexes were isolated as crystalline solids that were homogeneous under microscope inspection. The purity of metal complexes was readily assessed by a combination of ^1^H and ^19^F NMR spectroscopies. All Fe complexes formed homogeneous solutions in noncoordinating solvents, e.g., C_6_H_6_, were ^7^Li NMR silent and had no traces of coordinating solvents (e.g., THF) present by NMR analysis, thus precluding the presence of inorganic salts.

### Spectroscopy and Spectrometry

NMR spectra were recorded on a Bruker 300 or 400 MHz spectrometer. ^1^H and ^13^C chemical shifts are reported in ppm relative to tetramethylsilane using residual solvent as an internal standard. ^19^F chemical shifts are reported in ppm relative to 5% v/v internal PhF.^46^ Solution-phase effective magnetic moments were determined by the method described by Evans^47^ and are corrected for diamagnetic contributions.^48^ Mass spectrometry data were collected on a Bruker micrOTOF II with an ESI source. FTIR spectra were recorded on solid or solution samples using a Bruker Alpha II FTIR spectrometer operating at 4 cm^–1^ resolution. The magnetic susceptibility of [(^tBu^**L**)Fe(μ_2_-H)]_2_ was measured on a Johnson Matthey Mark I balance.

### X-ray Crystallography

Low-temperature diffraction data were collected on a Bruker AXS X8 Kappa Duo diffractometer coupled to an APEX2 CCD detector. The data collections were executed with Mo Kα radiation (λ = 0.71073 Å) from a IμS microsource, performing ϕ and ω scans. Absorption and other corrections were applied using the program *SADABS*.^49,50^ The structures were solved by dual-space methods using *SHELXT*(51) and refined against *F*^2^ on all data by full-matrix least-squares with *SHELXL-2017*,^52^ following established refinement strategies.^53^ All non-hydrogen atoms were refined anisotropically, and all hydrogen atoms not bound to Fe were included into the model at geometrically calculated positions and refined using a riding model. Those hydrogen atoms bound to Fe were allowed to refine freely. The structure of (^tBu^**L**)FeI contained a highly disordered molecule of *n*-pentane for which no interpretable electron density maxima could be found; this was treated with the program *SQUEEZE*.^54^

### Computational Details

DFT calculations carried out using revision 5.0.3 of the *ORCA* suite of programs,^55^ using the “TIGHTSCF” convergence criteria and default settings otherwise.

Unless otherwise noted, all DFT calculations made use of the TPSS meta-GGA exchange-correlation functional including 10% exact Hartree–Fock exchange (TPPSh)^56−58^ with the def2-TZVP basis set.^59^ Additional calculations made use of the BP86^60^ and B3LYP^61^ functionals. Calculations were accelerated through the RIJCOSX approximation.^62^ A truncated model of ^R^**L** (*L) was constructed by replacing the tBu/Ad pyrazole substituents with −CH_3_. This was anticipated to qualitatively model the donor properties of ^R^**L** while reducing computational cost.^63^ Initial geometries for all Fe complexes were taken from the crystallographically determined coordinates with the positions of all H atoms allowed to relax along the ground-state potential energy surface. As an exception, to calculate the relative energies of the high (*S* = 2) and intermediates (*S* = 1) spin states for (*L)FeH, all atomic coordinates were left unconstrained. The contracted auxiliary Coulomb fitting basis def2/J was applied to all atoms.^64^ Grimme’s atom-pairwise correction with Becke-Johnson damping (D3BJ) was included to account for the effects of dispersion.^65^

For single-point calculations on [(*L)Fe(μ_2_-H)]_2_, we generated a low-spin, broken-symmetry (BS) determinant by first converging a high-spin determinant (*M*_S_ = 4). The α and β spin density matrix elements on the Fe sites were then exchanged via the FlipSpin feature of ORCA and a low-spin determinant reconverged (*M*_S_ = 0). The exchange-coupling constant (*J*) was estimated as −85 cm^–1^ using the following formula:^66^
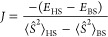


Mössbauer parameters were computed using the “eprnmr” module of *ORCA* with the radial integration accuracy about the Fe increased (IntAcc7).^39^ Only molecular orbitals greater in energy than the Fe(3s) orbital were localized; these were constructed using the intrinsic bond orbital method developed by Knizia.^67^ Isomer shifts in mm s^–1^ were determined from calculated core electron densities (ρ_0_) according to the empirical formulas determined by Neese.^43^

### Safety Statement

***Caution!** tBuLi is extremely pyrophoric! The risks associated with this useful reagent are largely mitigated by limiting its use to inside an N_2_ glovebox, as we report below. No other uncommon hazards are noted.*

### Synthetic Procedures

#### K[DAl(OtBu)_3_]

The initial synthesis of Li[DAl(OtBu)_3_] is adapted from the literature.^68^ The overall synthesis is suitable for the preparation of both K[DAl(OtBu)_3_] and K[HAl(OtBu)_3_]. Under an N_2_ atmosphere, Li[AlD_4_] (0.500 g, 0.0132 mol) was dissolved in Et_2_O (30 mL), and tBuOH (4.0 mL, 0.042 mol) added dropwise via a syringe. *Note:* The Li[AlD_4_] *must* be purified before use by dissolving in Et_2_O, filtering over Celite and removing volatiles under reduced pressure; the tBuOH *must* also be distilled from CaH_2_ prior to use. Addition of the tBuOH causes vigorous, but easily managed, effervescence of D_2_. Toward the end of the addition Li[DAl(OtBu)_3_] precipitates from solution as a colorless solid. To prevent contamination of the glovebox atmosphere with tBuOH, volatiles were thoroughly stripped from the reaction mixture and the residue dried at 60 °C under reduced pressure for ∼3 h. The reaction flask was taken into the glovebox, the solids resuspended in Et_2_O (20 mL) and collected by filtration. Yield: 2.30 g, 70%. A portion of this material was used in the next step. Li[DAl(OtBu)_3_] (2.00 g, 7.80 mmol) was suspended in C_6_H_6_ (10 mL) and KOtBu (0.880 g, 7.80 mmol) added as a suspension in C_6_H_6_ (5 mL) via pipet. An additional 5 mL of C_6_H_6_ was used to wash any remaining residue of KOtBu into the reaction flask. The reaction mixture was then stirred for 48 h to yield a colorless, oily solid suspended in the C_6_H_6_. THF (30 mL) was added to dissolve the suspended material and the mixture filtered through Celite to remove a small amount of insoluble solids. Volatiles were removed thoroughly under reduced pressure to afford tacky white solids; these were suspended in Et_2_O (20 mL) and collected by filtration. The solid was washed well with Et_2_O (3 × 10 mL) then pentane (3 × 5 mL). Yield: 2.09 g, 93%. This material was sufficiently pure for our purposes, but could be recrystallized with ∼80% recovery by dissolving in THF (10 mL per 1g solid), diluting with 4 volumes of Et_2_O and cooling to −30 °C for several hours. Spectroscopic data for the hydride are as follows, with spectra for both hydride and deuteride provided in the SI. ^1^H NMR (300 MHz, CD_3_CN): δ 1.17 (s, OC(C*H*_3_)_3_) (the broad *H*–Al resonance could not be readily identified). ^27^Al NMR (104 MHz, CD_3_CN): δ 74.94 (d, *J*_Al–H_ = 230 Hz). FTIR cm^–1^: 2962s, 2921m, 2894w, 2861m, 1719s (Al–H), 1456w, 1380w, 1355m, 1224m, 1203s, 1058w, 1024w, 991s, 913w, 782s, 695m, 642m, 584m, 488w, 451w, 440w, 421w.

#### 3-(Adamantan-1-yl)-5-(*tert*-butyl)-1H-pyrazole (^tBu,Ad^pzH)

To a stirred suspension of NaH (12.1 g, 0.505 mol) in THF (no predrying is required), a solution of 1-acetyladamantane (30.0 g, 0.170 mol) and methyl trimethyl acetate (39.1 g, 0.340 mol) in THF (50 mL) was added. The mixture was heated to 65 °C for 48 h to afford a brown solution with a small amount of residual NaH still visible. The suspension was cooled to room temperature (RT) and the remaining NaH quenched via dropwise addition of water. The mixture was made strongly acidic with HCl (6 M, ∼200 mL) and diluted with hexanes (200 mL). The organic layer was separated, washed with water (3 × 50 mL), dried over Na_2_SO_4_, filtered, and the solvent removed under reduced pressure. The resulting yellow-brown oil was dissolved in EtOH (300 mL), hydrazine monohydrate (32 g, 1.0 mol) added and the mixture heated at 85 °C for 3 h. The yellow reaction mixture was cooled and water (∼800 mL) added slowly to precipitate the crude pyrazole as a yellow solid. This solid was collected by filtration, washed with copious water and sucked dry. The crude product was dissolved in the minimum CH_2_Cl_2_ and diluted with three volumes of acetonitrile. This solution was heated to boiling until the CH_2_Cl_2_ had fully evaporated at which point the product precipitates as a cream solid. The mixture was cooled to RT and the pyrazole collected by filtration and washed with acetonitrile (3 × 50 mL). Yield: 27.4 g (63% over 2 steps). ^1^H NMR (400 MHz, CDCl_3_): δ 10.42 (s, 1H, N*H*), 5.88 (s, 1H, pz*H*), 2.07 (m, 3H, 3 × ^Ad^C*H*), 1.97 (m, 6H, 3 × ^Ad^C*H*_2_), 1.77 (m, 6H, 3 × ^Ad^C*H*_2_), 1.33 (s, 9H, C(C*H*_3_)_3_). ^13^C{^1^H} NMR (101 MHz, CDCl_3_): 158.58 (3/5-pz*C*), 157.33 (3/5-pz*C*), 96.78 (4-pz*C*), 42.81 (^Ad^*C*H_2_), 36.91 (^Ad^*C*H_2_), 33.44 (^Ad^*C*), 31.70 (*C*(CH_3_)_3_), 30.68 (C(*C*H_3_)_3_), 28.68 (^Ad^*C*H). ESI-MS(+): *m*/*z*: 259.2178; calc. for [^tBu,Ad^pzH+H]^+^: *m*/*z* 259.2174.

#### (^tBu,Ad^pz)_2_CH_2_

To a solution of ^tBu,Ad^pzH (37.6 g, 0.146 mol) and BzNEt_3_Cl (11 g, 0.048 mol) in CH_2_Cl_2_ (600 mL) and CH_2_Br_2_ (10 mL) was added NaOH (aq, 600 mL, 50% w/w). The biphasic mixture was stirred vigorously overnight at RT, then transferred to a separatory funnel and the lower aqueous layer discarded. The organic layer was diluted with an equal volume of hexanes washed with water (3 × 300 mL). The organic layer was then dried over Na_2_SO_4_, filtered, and the volatiles removed under reduced pressure to yield a tan solid that consisted of a regioisomeric mixture of methylene-bridged pyrazoles. The desired isomer was obtained as follows (this procedure is entirely reproducible if performed with care). The crude solid was first recrystallized from the minimum boiling methyl ethyl ketone. This yields a roughly 2:1 mixture of dissymmetric:desired regioisomers. This solid is then recrystallized twice from the minimum boiling hexanes to obtain the desired regioisomer in near pure form. Care must be taken to only cool the crystallizations to RT (∼25 °C; *not colder*), and to not leave these for prolonged periods to prevent undesired regioisomers from crystallizing out. A final, straightforward recrystallization is performed by dissolving the solid in the minimum CH_2_Cl_2_, diluting with 3 volumes of acetonitrile and boiling the solution until all the CH_2_Cl_2_ has evaporated. The resulting colorless crystals are collected by filtration and washed with acetonitrile (3 × 5 mL). Yield: 2.89 g (7.5%). The combined mother liquors can be combined, evaporated, and the starting pyrazole recovered quantitatively by boiling the crude solid in a mixture of concentrated HCl and MeOH overnight. ^1^H NMR (400 MHz, CDCl_3_): δ 6.57 (s, 2H, 2 × pz*H*), 5.93 (s, 2H, pz_2_C*H*_2_), 2.05 (m, 6H, 6 × ^Ad^C*H*), 1.93 (m, 12H, 6 × ^Ad^C*H*_2_), 1.78 (m, 12H, 6 × ^Ad^C*H*_2_), 1.21 (s, 18H, 2 × C(C*H*_3_)_3_). ^13^C{^1^H} NMR (101 MHz, CDCl_3_): δ 159.21 (3/5-pz*C*), 152.90 (3/5-pz*C*), 101.65 (4-pz*C*), 67.34 (pz_2_*C*H_2_), 42.58 (^Ad^*C*H_2_), 36.97 (^Ad^*C*H_2_), 33.83 (^Ad^*C*), 31.97 (*C*(CH_3_)_3_), 29.65 (C(*C*H_3_)_3_), 28.72 (^Ad^*C*H). ESI-MS(+): *m*/*z*: 529.4257; calc. for [^tBu,Ad^pz_2_CH_2_+H]^+^: *m*/*z* 529.4270.

#### ^Ad^**L**H

In the glovebox (^tBu,Ad^pz)_2_CH_2_ (0.99 g, 0.0019 mol) was dissolved in THF (8 mL), cooled to −78 °C and nBuLi (2.5 M, 0.90 mL, 0.0023 mol) added slowly via a syringe. This mixture was stirred at −78 °C for 6 h at which point SiMe_2_Cl_2_ (0.29 mL, 0.0032 mol) was added to the solution dropwise via a syringe. The resulting solution was removed from the cold well and allowed to warm to RT. After stirring an additional 5 min, volatiles were thoroughly removed under vacuum to yield a white solid. This material was sufficiently pure for the next step. The crude solid was dissolved/suspended in Et_2_O (∼4 mL), and an Et_2_O (∼8 mL) solution of (3,5-(CF_3_)_2_C_6_H_3_)CH_2_MgCl added slowly via pipet at RT. The Grignard reagent was prepared from (3,5-(CF_3_)_2_C_6_H_3_)CH_2_Cl (0.68 g, 0.0028 mol) according to our previously reported procedure.^30^ The reaction was left to stir for 2 h then removed from the glovebox and quenched by careful dropwise addition of water. The organic layer was separated, dried over Na_2_SO_4_, and solvent removed under reduced pressure to yield a yellow oil. CH_3_OH (3 mL) and a small stir bar were added and the mixture stirred rapidly for 3 h to yield a white solid and a yellow supernatant. The solid was collected by filtration and washed with CH_3_OH (3 × 3 mL). Yield: 1.19 g (78% over 2 steps). ^1^H NMR (400 MHz, CDCl_3_): δ 7.59 (m, 3H, 2 × *o*-Ar^F^*H* + *p*-Ar^F^*H*), 6.85 (s, 1H, pz_2_C*H*), 5.91 (s, 2H, 2 × pz*H*), 2.47 (s, 2H, C*H*_2_–Ar^F^), 2.06 (m, 6H, 6 × ^Ad^C*H*), 1.94 (m, 12H, 6 × ^Ad^C*H*_2_), 1.78 (m, 12H, 6 × ^Ad^C*H*_2_), 1.06 (s, 18H, 2 × C(C*H*_3_)_3_), 0.14 (s, 6H, 2 × Si(CH_3_)_2_). ^13^C{^1^H} NMR (101 MHz, CDCl_3_): δ 158.58 (Ar*C*), 153.65 (Ar*C*), 143.84 (Ar*C*), 131.31 (q, *J*_CF_ = 30 Hz, *m*-Ar^F^*C*), 128.53 (m, *o*-Ar^F^*C*), 123.76 (q, *J*_CF_ = 270 Hz, *C*F_3_), 118.13 (sept, *J*_CF_ = 4 Hz, *p*-Ar^F^*C*), 101.94 (Ar*C*), 73.82 (pz_2_-*C*H), 42.81 (^Ad^*C*H_2_), 37.11 (^Ad^*C*H_2_), 33.95 (^Ad^*C*), 32.25 (*C*(CH_3_)_3_), 30.32 (C(*C*H_3_)_3_), 28.86 (^Ad^*C*H), 25.66 (*C*H_2_–Ar^F^), −1.44 (Si(*C*H_3_)_2_). ^19^F NMR (376 MHz, CDCl_3_): δ −62.66 (s, C*F*_3_). ESI-MS(+): *m*/*z* 813.4681; calc. for [^Ad^**L**H+H]^+^: *m*/*z* 813.4726.

#### (^tBu^**L**)FeI

A stirred solution of ^tBu^**L**H (0.314 g, 0.490 mmol) was cooled to −78 °C in the glovebox coldwell and tBuLi (2.7 M, 0.20 mL, 0.54 mmol) added dropwise via a syringe. The reaction vessel was then transferred to the −30 °C glovebox freezer and left standing for 2 h to ensure complete deprotonation. While the mixture was still cold, FeI_2_(THF)_2.67_ (0.222 g, 0.588 mmol) was added as a solid with stirring. The reaction was allowed to come to RT and stirred an additional hour. Volatiles were then removed under reduced pressure to yield a dark yellow, oily solid. Pentane (∼5 mL) was added, which caused the oil to solidify. The crude, yellow solid was collected by filtration and washed with additional pentane (3 × 5 mL). The solid was suspended in Et_2_O (4 mL), stirred rapidly for 30 min, and again collected by filtration and washed with Et_2_O (3 × 2 mL). This process removes LiI. The solid was dissolved in THF (∼5 mL), diluted with one volume of pentane, and this solution eluted through a 5 cm pad of silica on a 20 mL glass fritted funnel. The silica was washed with further 1:1 THF–pentane until the eluent was colorless. This removes residual FeI_2_(THF)_2.67_. The success of this chromatography can be assessed by removing the silica column from the glovebox and allowing adsorbed material to oxidize. The bottom portion of the silica should remain entirely colorless. Volatiles were removed from the combined fractions under reduced pressure to yield a yellow crystalline solid. This was suspended in Et_2_O (∼5 mL) and 2 volumes of pentane added to complete precipitation. The pure product was collected on a glass frit and washed with pentane (3 × 3 mL). Yield: 0.242 (59%). X-ray quality crystals were grown by layering an Et_2_O solution with pentane at −30 °C. RT magnetic moment (by Evans method in C_6_D_6_): 5.2 μ_B_. ^1^H NMR (400 MHz, C_6_D_6_): δ 83.07 (1H), 68.50 (1H), 66.08 (1H), 15.22 (9H, C(C*H*_3_)_3_), 6.12 (9H, C(C*H*_3_)_3_), −7.40 (3H, Si(C*H*_3_)), −30.25 (9H, C(C*H*_3_)_3_), −33.73 (9H, C(C*H*_3_)_3_), −48.29 (3H, Si(C*H*_3_)), −92.28 (1H). Resonances for 2 × *o*-Ar^F^*H* and (presumably) Fe–C*H* not observed. ^19^F NMR (376 MHz, C_6_D_6_): δ −91.24 (s, C*F*_3_). FTIR cm^–1^: 3012w, 2964m, 2901s, 2847m, 2359m, 2334m, 1651w, 1597w, 1538w, 1454w, 1416s, 1365w, 1313m, 1291s, 1273m, 1254m, 1236m, 1200m, 1162s, 1125s, 1101m, 1085w, 1021w, 995w, 947m, 880w, 851w, 836w, 807w, 744w, 729w, 701w, 680w, 667w, 652w. UV–vis (C_6_H_6_; λ_max_ (nm) ε_max_ (cm^–1^ M^–1^): 297 (7.8 × 10^3^), 337 (7.0 × 10^3^), 430sh.

#### [(^tBu^**L**)Fe(μ_2_-H)]_2_

(^tBu^**L**)FeI (0.050 g, 0.0060 mmol) was dissolved/suspended in Et_2_O (4 mL) and K[HAl(OtBu)_3_] (0.050 g, 0.17 mmol) added as a solid with stirring. The mixture was left to stir at RT for precisely 1 h. At this point colorless solids were removed by filtration through a short pad of Celite. Volatiles were then removed under reduced pressure to yield an oily orange solid. Pentane (2 mL) was added and the mixture cooled to −30 °C in the glovebox freezer for several hours to yield a dark-orange, crystalline solid. Pentane was decanted from the crystals, which were washed with additional pentane (2 mL). This material required further purification to remove a small paramagnetic impurity: the crystals were dissolved in *o*-DFB (6 mL) with stirring, filtered over a short pad of Celite and concentrated to ∼2 mL. This solution was diluted with 6 volumes of pentane and placed in the −30 °C glovebox freezer overnight to yield large orange crystals of the product. Yield: 0.026 g (61%). X-ray quality crystals were grown by cooling a saturated Et_2_O solution layered with pentane of the complex to −30 °C overnight. RT magnetic moment (by Evans method in C_6_D_6_): 5.1 μ_B_ (per Fe); in the solid state: 5.0 ± 0.2 μ_B_ (per dimer). ^1^H NMR (400 MHz, C_6_D_6_) (peaks for (^tBu^**L**)FeH; see main text): δ 86.17 (1H), 73.46 (1H), 59.39 (1H), 17.35 (9H, C(C*H*_3_)_3_), 11.89 (9H, C(C*H*_3_)_3_), 7.51 (3H, Si(C*H*_3_)), −24.64 (3H, Si(C*H*_3_)), −39.39 (9H, C(C*H*_3_)_3_), −40.66 (9H, C(C*H*_3_)_3_), −87.80 (1H). Resonances for 2 × *o*-Ar^F^*H* and (presumably) Fe–C*H* and Fe–*H* not observed. ^19^F NMR (376 MHz, C_6_D_6_): δ −90.65 (s, C*F*_3_). FTIR cm^–1^: 2961s, 2904m, 1596m, 1537m, 1469m, 1416w, 1364s, 1288w, 1272s, 1243w, 1218m, 1207w, 1161s, 1123s, 1097m, 1056w, 993w, 945w, 889m, 849m, 745w, 727w, 702w, 680w, 650w, 561w, 443w, 419w. UV–vis (C_6_H_6_; λ_max_ (nm) ε_max_ (cm^–1^ M^–1^): 342 (5.5 × 10^3^), 451sh.

#### (^Ad^**L**)FeI

This complex was prepared in an essentially identical fashion to (^tBu^**L**)FeI. (^Ad^**L**)FeI obtained via this method, while quite pure, was contaminated with a small amount of a paramagnetic impurity that needed to be removed prior to the synthesis of (^Ad^**L**)FeH. This could be reproducibly performed as follows: crude (^Ad^**L**)FeI was dissolved in the minimum of Et_2_O (∼15 mL per 150 mg), filtered through a small piece of tissue and the solution concentrated to roughly half the original volume. The solution was then placed into the glovebox freezer at −30 °C overnight to yield a yellow crystalline solid. Et_2_O was decanted off the crystals which were then washed with pentane (3 × 5 mL). Yield: 0.373 g (61%) from 0.920 g of ^Ad^**L**H. X-ray quality crystals were grown by layering an Et_2_O solution of the complex with pentane at −30 °C overnight. RT magnetic moment (by Evans method in C_6_D_6_): 5.4 μ_B_. ^1^H NMR (400 MHz, C_6_D_6_): δ 80.96 (1H), 72.75 (1H), 64.63 (1H), 14.67 (9H, C(C*H*_3_)_3_), 5.62 (9H, C(C*H*_3_)_3_), −5.43 (3H), −6.37 (3H), −7.84 (3H), −8.89 (3H), −9.27 (3H), −12.59 (3H), −16.65 (3H), −29.62 (3H), −32.31 (6H), −41.44 (3H), −50.92 (3H), −89.15 (1H). The expected 10 + 2 peaks for the 1-adamantyl and Si(CH_3_)_2_ protons are observed. Resonances for 2 × *o*-Ar^F^*H* and (presumably) Fe–C*H* not observed. ^19^F NMR (376 MHz, C_6_D_6_): δ −91.31 (s, C*F*_3_). FTIR cm^–1^: 3012w, 2964m, 2901s, 2847w, 2359w, 2334w, 1538w, 1454w 1416w, 1365w, 1313m, 1291s, 1273w, 1254w, 1236w, 1200w, 1162w, 1125w, 1101s, 1085s, 1052w, 1021w, 995w, 947w, 880m, 851w, 836w, 807w, 744w, 729w, 701w, 680w, 667w, 652w. UV–vis (C_6_H_6_; λ_max_ (nm) ε_max_ (cm^–1^ M^–1^): 299 (9.9 × 10^3^), 340 (8.7 × 10^3^), 430sh.

#### (^Ad^**L**)FeH

(^Ad^**L**)FeI (0.200 g, 0.020 mmol) was dissolved/suspended in Et_2_O (10 mL), K[HAl(OtBu)_3_] (0.56 g, 2.0 mmol) added as a solid, and the mixture left to stir at RT for precisely 14 h. At this point colorless solids were removed by filtration through a short pad of Celite. Volatiles were then removed under reduced pressure to yield a golden yellow, oily solid. This was stirred with pentane (∼4 mL) to yield a crystalline yellow solid that was collected by filtration and washed with pentane (3 × 5 mL). The crude solid was dissolved in the minimum THF (∼1 mL), this solution diluted with pentane (∼10 mL) and quickly filtered through a short pad of Celite. Cooling to −30 °C in the glovebox freezer overnight yielded yellow crystals of the product as the pentane solvate; these were collected by filtration and washed with pentane (3 × 3 mL). Yield: 0.126 g (72%). X-ray quality crystals were grown by layering a saturated toluene solution with [(CH_3_)_3_SiO]_2_ at −30 °C for several days. RT magnetic moment (by Evans method in C_6_D_6_): 5.3 μ_B_. ^1^H NMR (400 MHz, C_6_D_6_): δ 84.97 (1H), 71.46 (1H), 58.35 (1H), 17.31 (9H, C(C*H*_3_)_3_), 12.71 (9H, C(C*H*_3_)_3_), 8.20 (3H), 2.74 (3H), 2.69 (3H), 1.75 (3H), 1.44 (3H), −3.07 (3H), −5.61 (3H), −25.09 (3H), −50.85 (3H), −51.68 (3H), −59.84 (3H), −62.13 (3H), −87.15 (1H). The expected 10 + 2 peaks for the 1-adamantyl and Si(CH_3_)_2_ protons are observed. Resonances for 2 × *o*-Ar^F^*H* and (presumably) Fe–C*H* and Fe–*H* not observed. ^19^F NMR (376 MHz, C_6_D_6_): δ −90.51 (s, C*F*_3_). FTIR cm^–1^: 2967m, 2900s, 2846m, 1596m, 1576m (Fe–H), 1537w, 1450m, 1365s, 1313m, 1289m, 1272s, 1237w, 1200s, 1161s, 1124w, 1057w, 993m, 947m, 876w, 850w, 807w, 741w, 702w, 680w, 650w. UV–vis (C_6_H_6_; λ_max_ (nm) ε_max_ (cm^–1^ M^–1^): 300 (1.2 × 10^4^), 343 (8.8 × 10^3^), 430sh.

## Supporting Information Available

Cartesian coordinates (XYZ)
Experimental details and procedures and spectroscopic data for all new compounds (PDF)

## Supplementary Material

ic4c01596_si_001.xyz
