# Supplementary material for: Low-Coordinate Iron Hydride Chemistry at an N,N,C-Heteroscorpionate Platform
Source: Inorg Chem. 2024 Jul 22;63(31):14449–58. doi: 10.1021/acs.inorgchem.4c01596 (PMC11304392; doi:10.1021/acs.inorgchem.4c01596)
Supplement: Supplementary file 2 — ic4c01596_si_002.pdf [file ic4c01596_si_002.pdf]

Supporting Information for:

Low-coordinate Fe-Hydride Chemistry at an N,N,C Heteroscorpionate Platform

Addison Fraker,[1] Brittany N. Linn[2] and Alex McSkimming,[1]*

[1]Department of Chemistry, Tulane University, New Orleans, LA 70118.

[2]Department of Chemistry, Massachusetts Institute of Technology, Cambridge, MA 02139

*amcskimming@tulane.edu

Contents

Spectroscopic Data	S3
Additional Data	S20

Spectroscopic Data

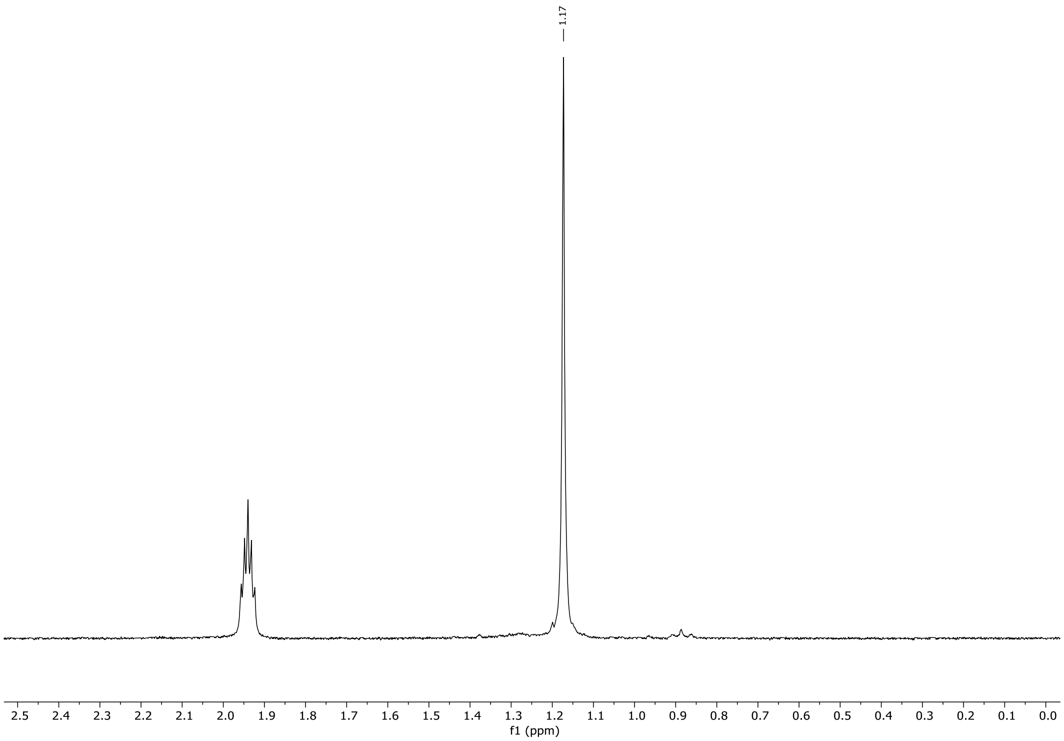

Figure S1. ^{1}H NMR spectrum of K[HAl(OtBu)$_3$] in CD_3CN at 300 MHz.

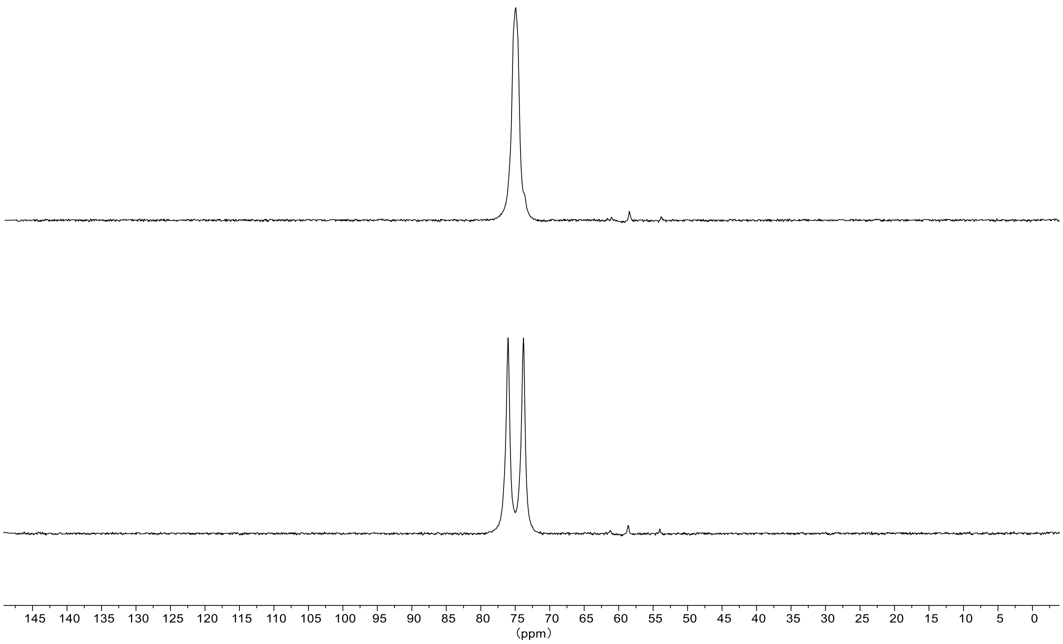

Figure S2. ^{27}Al NMR spectrum of K[DAl(OtBu)$_3$] (top) and K[HAl(OtBu)$_3$] (bottom) in CD_3CN at 104 MHz.

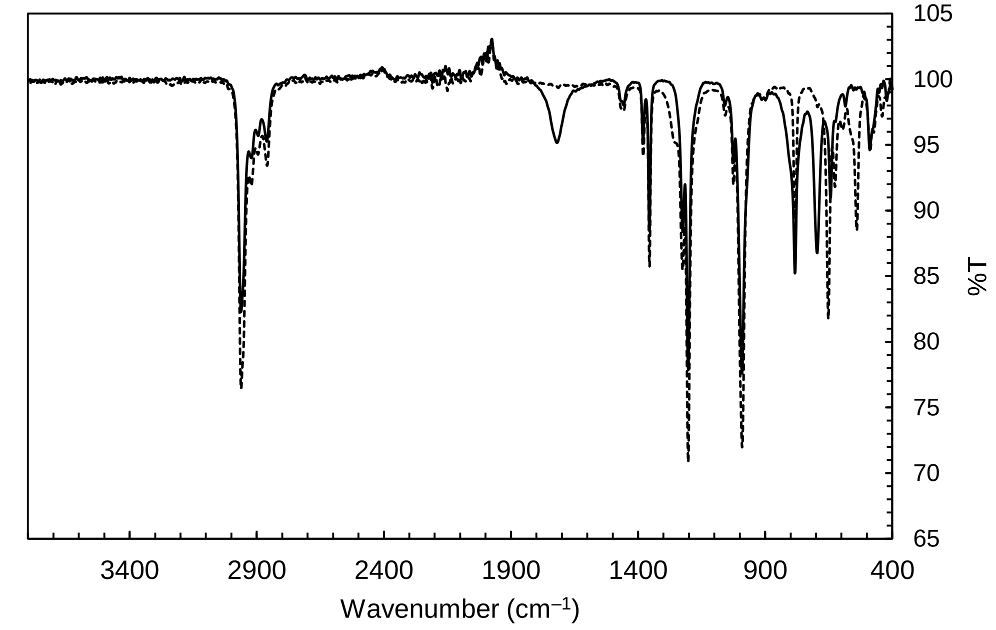

Figure S3. FTIR spectra of K[HAl(OtBu)$_3$] (solid line) and K[DAl(OtBu)$_3$] (dashed line).

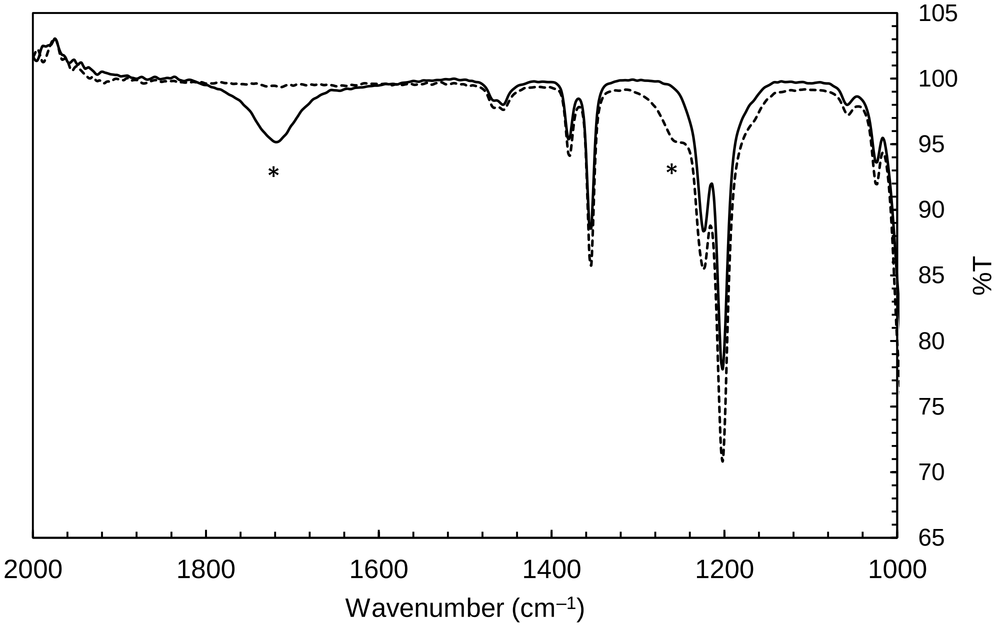

Figure S4. The above spectra; close-up. * mark the Al–H/D stretching bands.

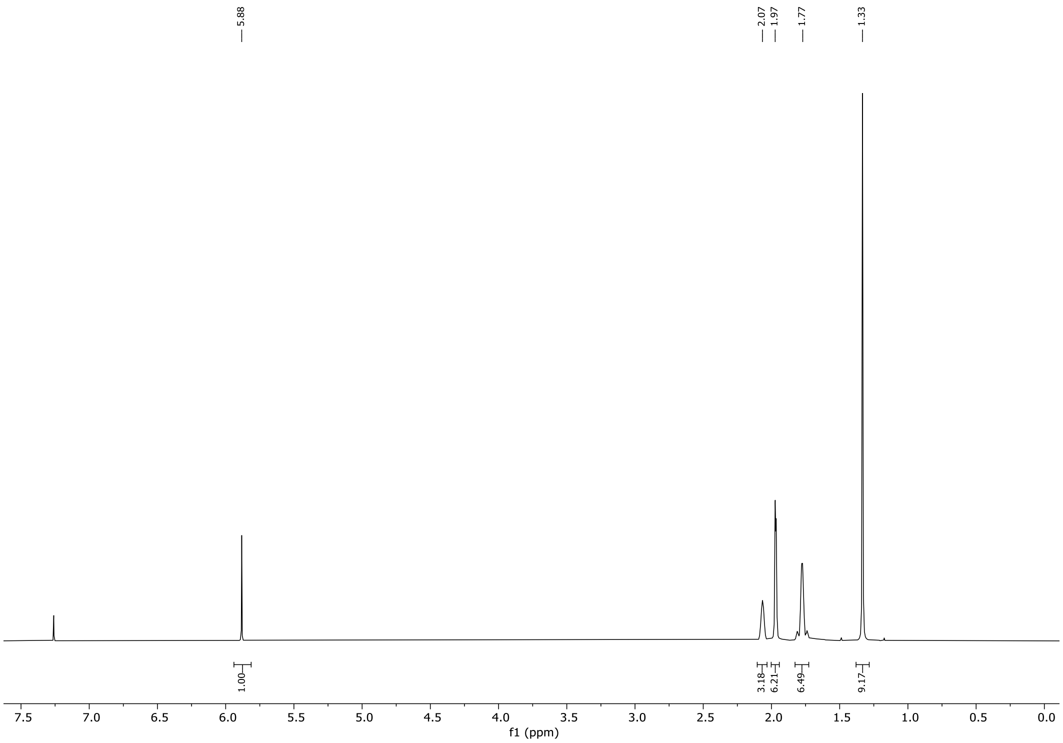

Figure S5. ^{1}H NMR spectrum of ^{tBu,Ad}pzH in $CDCl_3$ at 400 MHz.

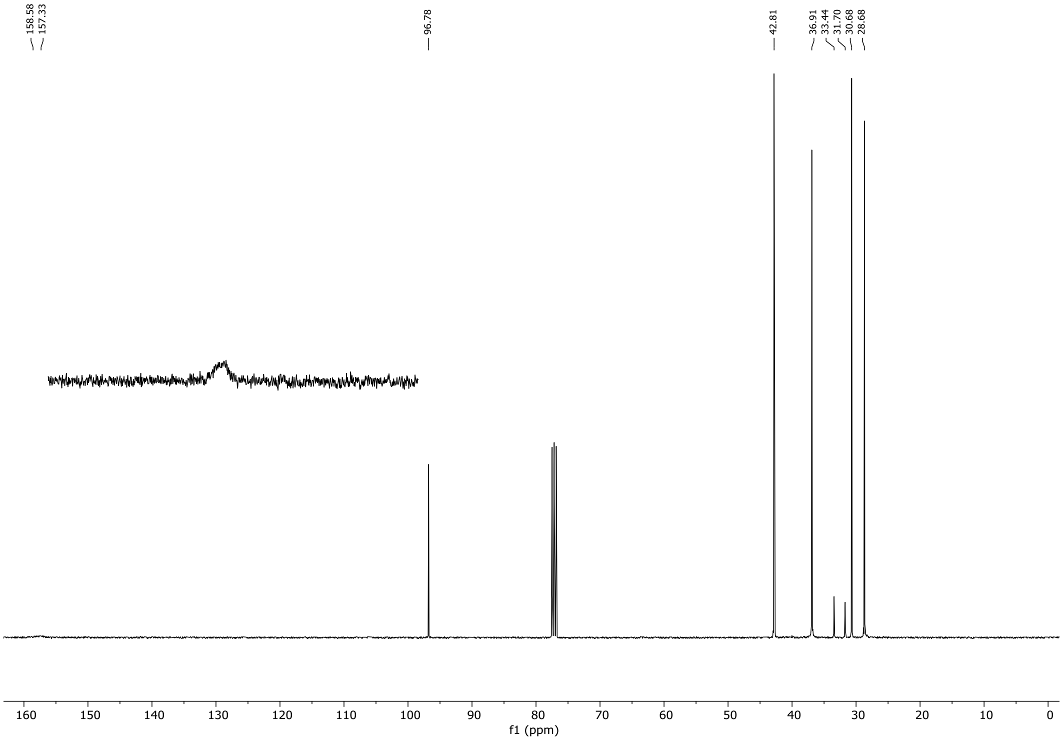

Figure S6. $^{13}C\{^{1}H\}$ NMR spectrum of ^{tBu,Ad}pzH in $CDCl_3$ at 101 MHz. Downfield region, inset.

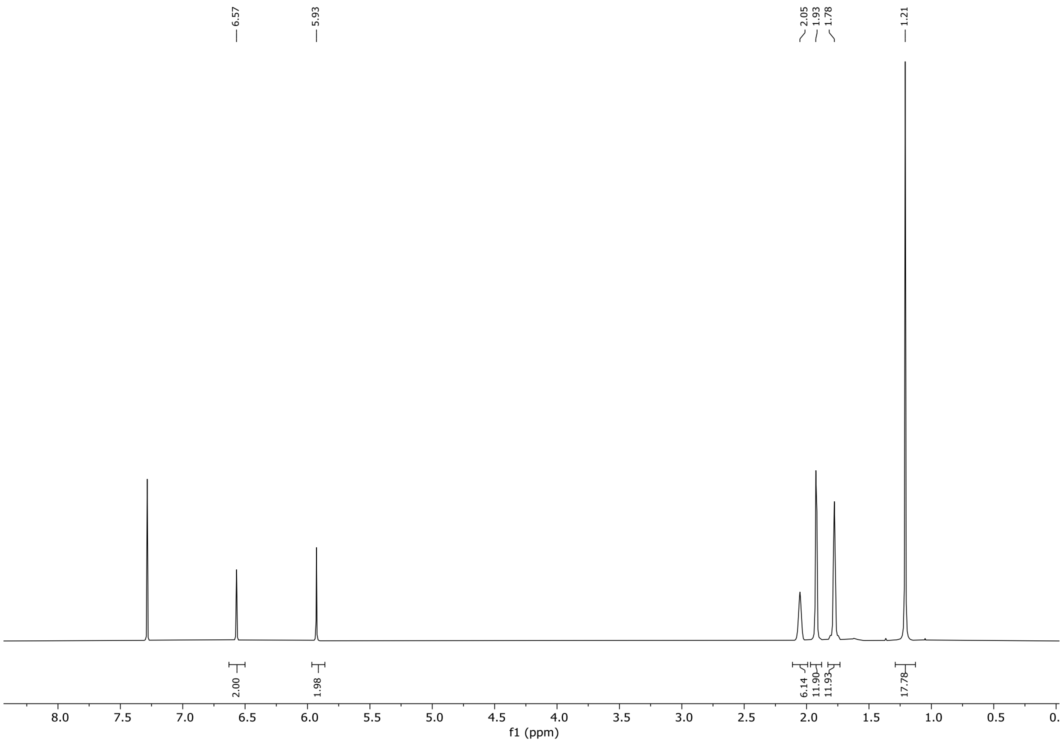

Figure S7. ^{1}H NMR spectrum of $(^{tBu,Ad}pz)_2CH_2$ in $CDCl_3$ at 400 MHz.

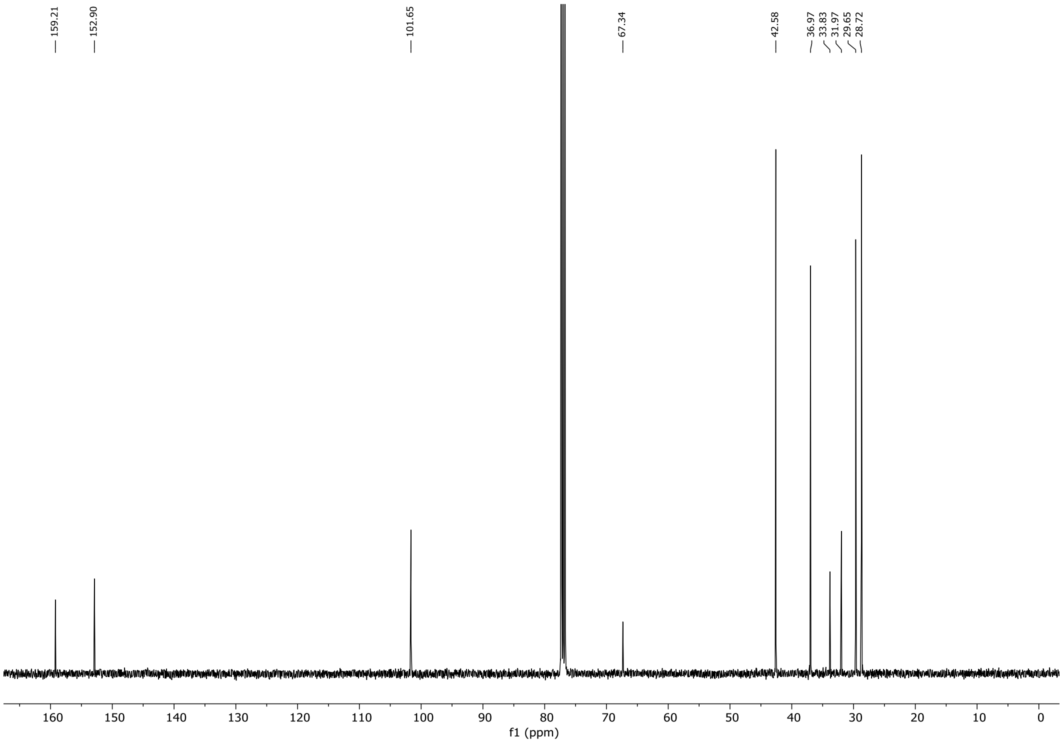

Figure S8. $^{13}C\{^{1}H\}$ NMR spectrum of $(^{tBu,Ad}pz)_2CH_2$ in $CDCl_3$ at 101 MHz.

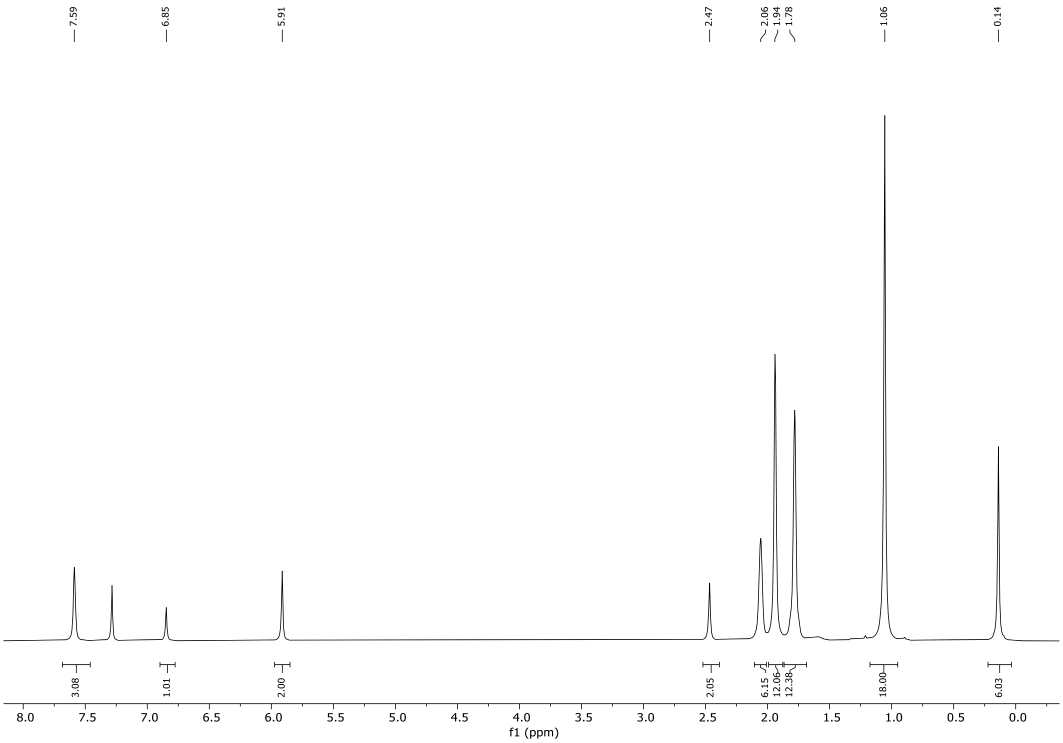

Figure S9. ^{1}H NMR spectrum of ^{Ad}**L**H in $CDCl_3$ at 400 MHz.

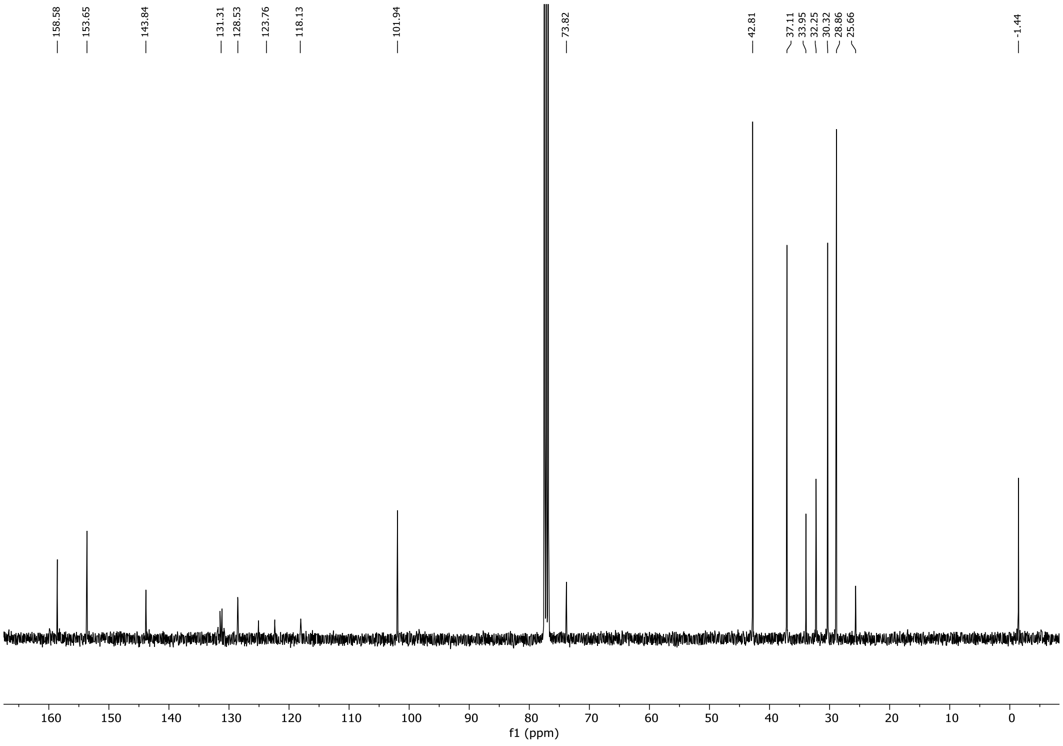

Figure S10. $^{13}C\{^{1}H\}$ NMR spectrum of ^{Ad}**L**H in $CDCl_3$ at 101 MHz.

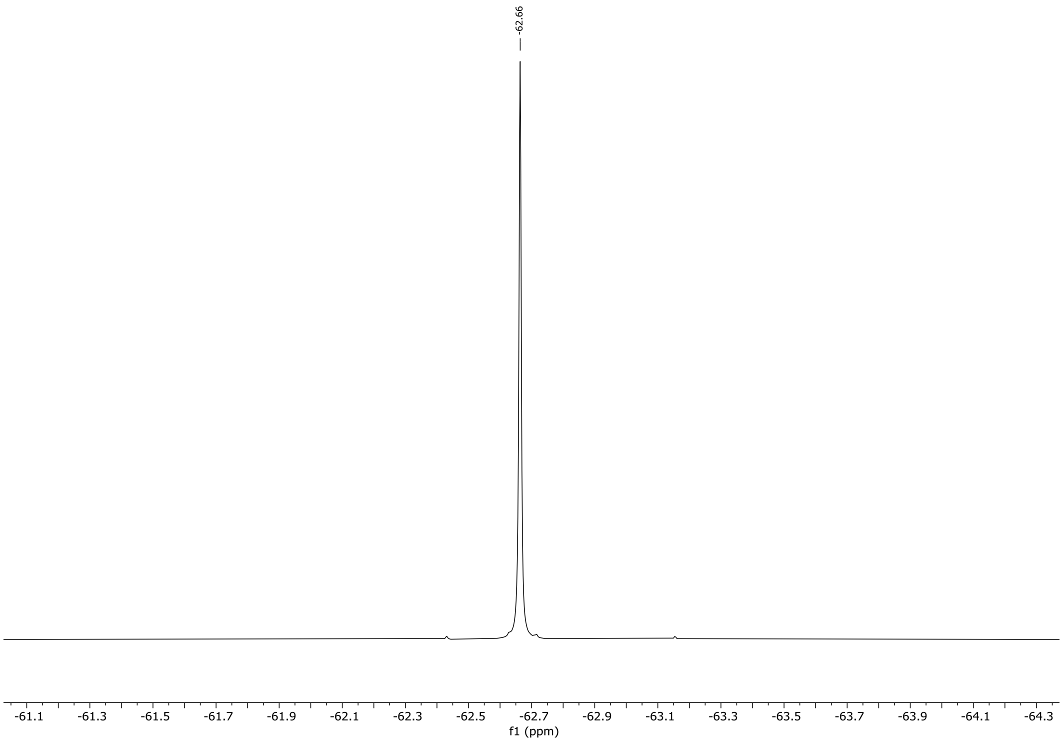

Figure S11. ^{19}F NMR spectrum of ^{Ad}**L**H in $CDCl_3$ at 376 MHz.

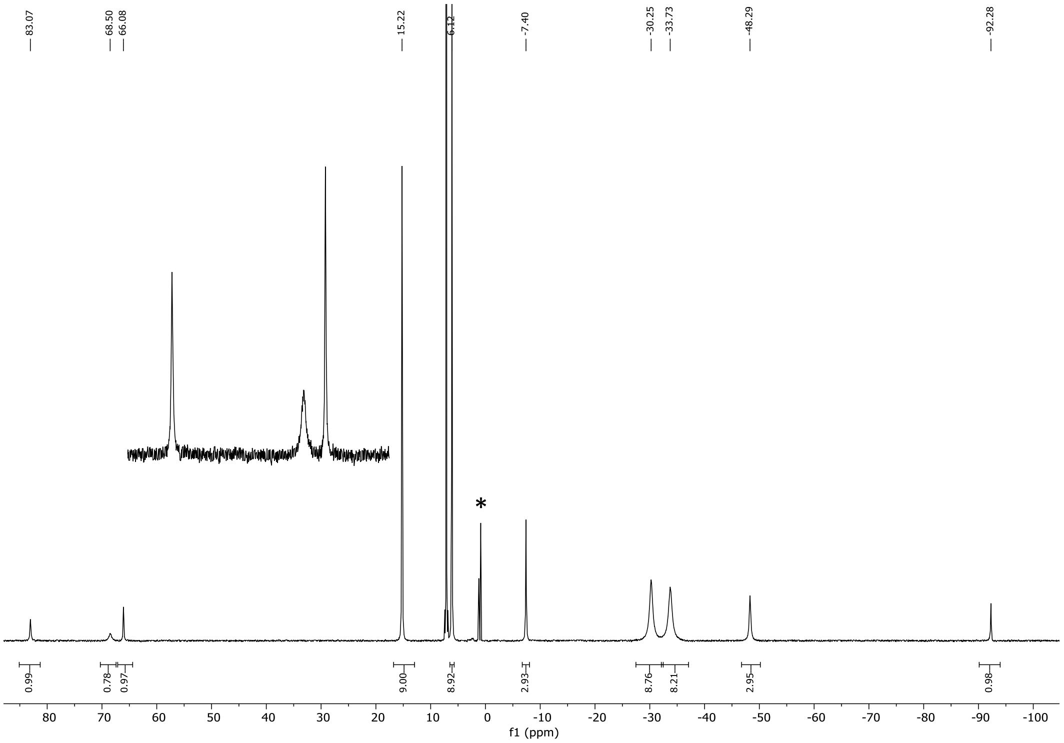

Figure S12. ^{1}H NMR spectrum of (^{tBu}**L**)FeI in C_6D_6 at 400 MHz. Downfield region, inset. * Marks n-pentane of crystallization.

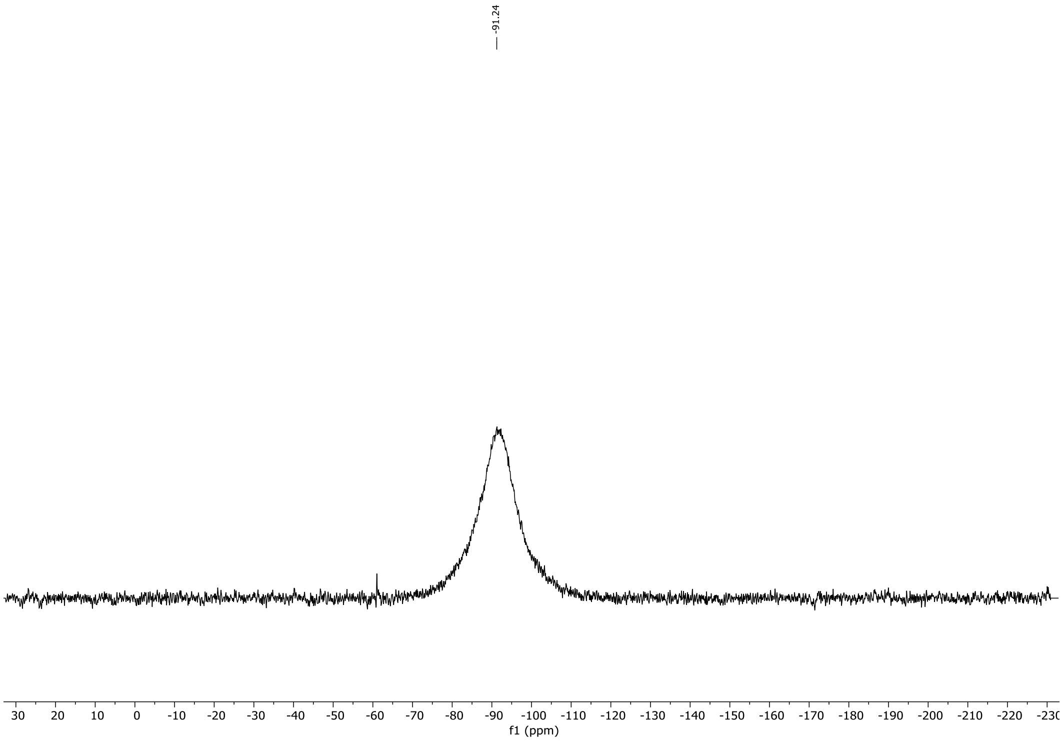

Figure S13. ^{19}F NMR spectrum of (^{tBu}**L**)FeI in C_6D_6 at 376 MHz.

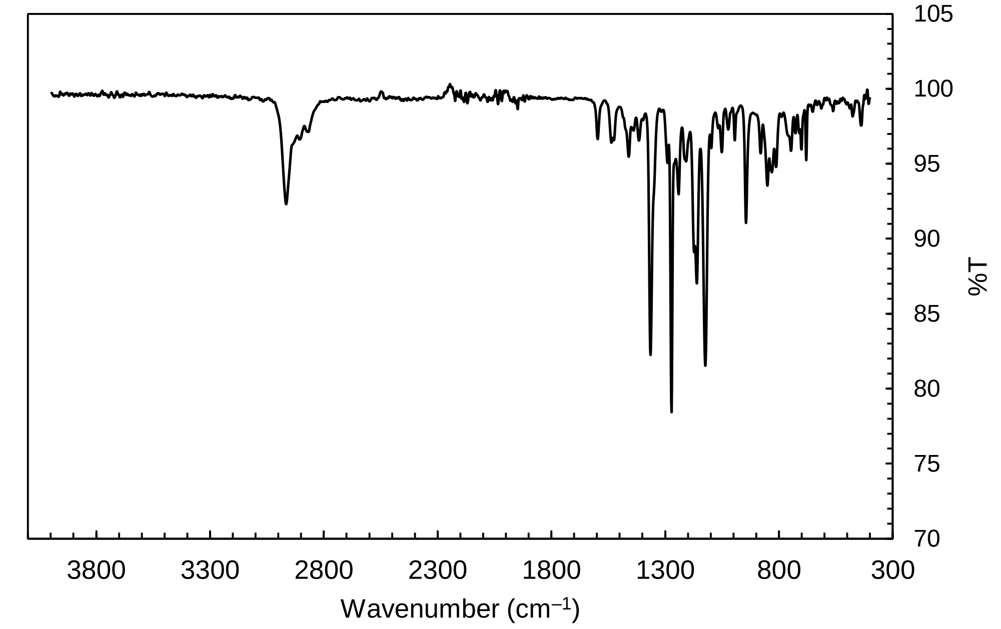

Figure S14. FTIR spectrum of (^{tBu}**L**)FeI.

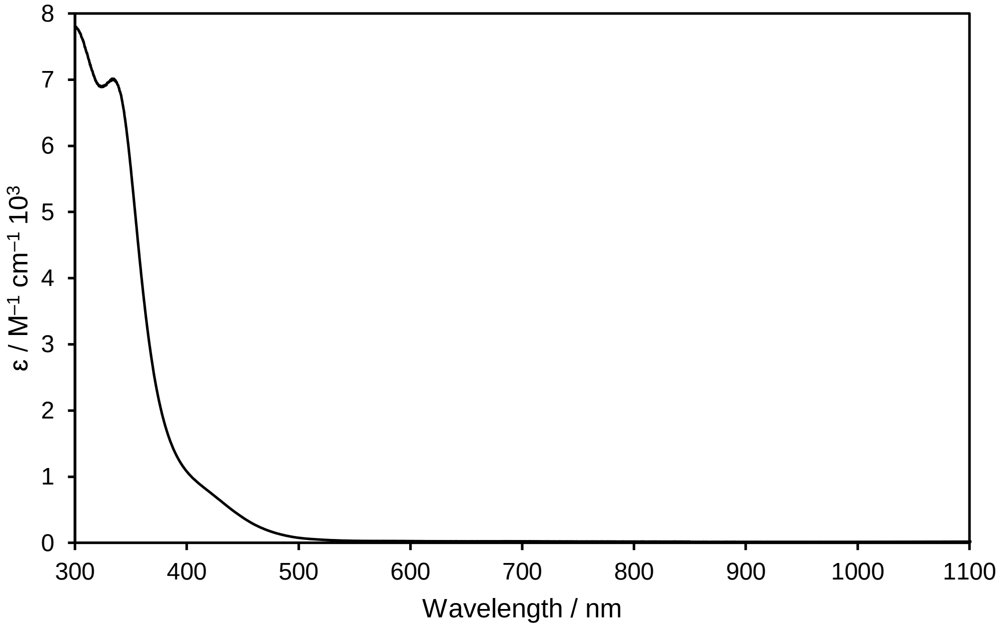

Figure S15. UV-Vis spectrum of (^{tBu}**L**)FeI in C_6H_6.

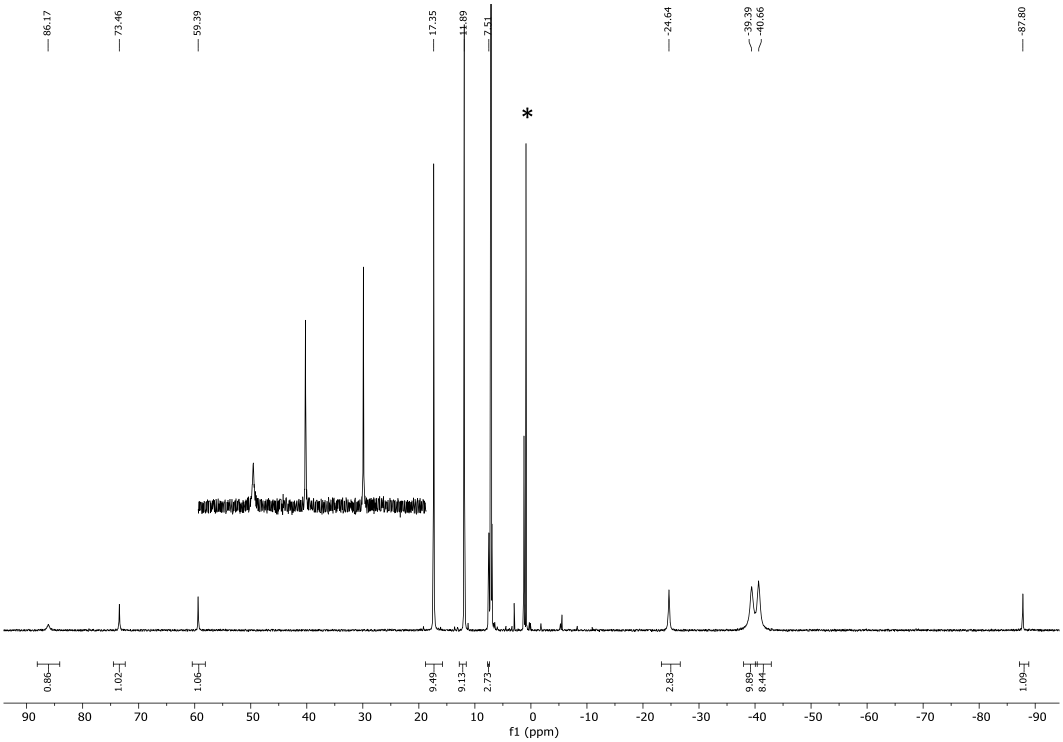

Figure S16. ^{1}H NMR spectrum of $[(^{tBu}\mathbf{L})Fe(\mu_2\text{-}H)]_2$ in C_6D_6 at 400 MHz. Downfield region, inset. * Marks peaks due to n-pentane of crystallization.

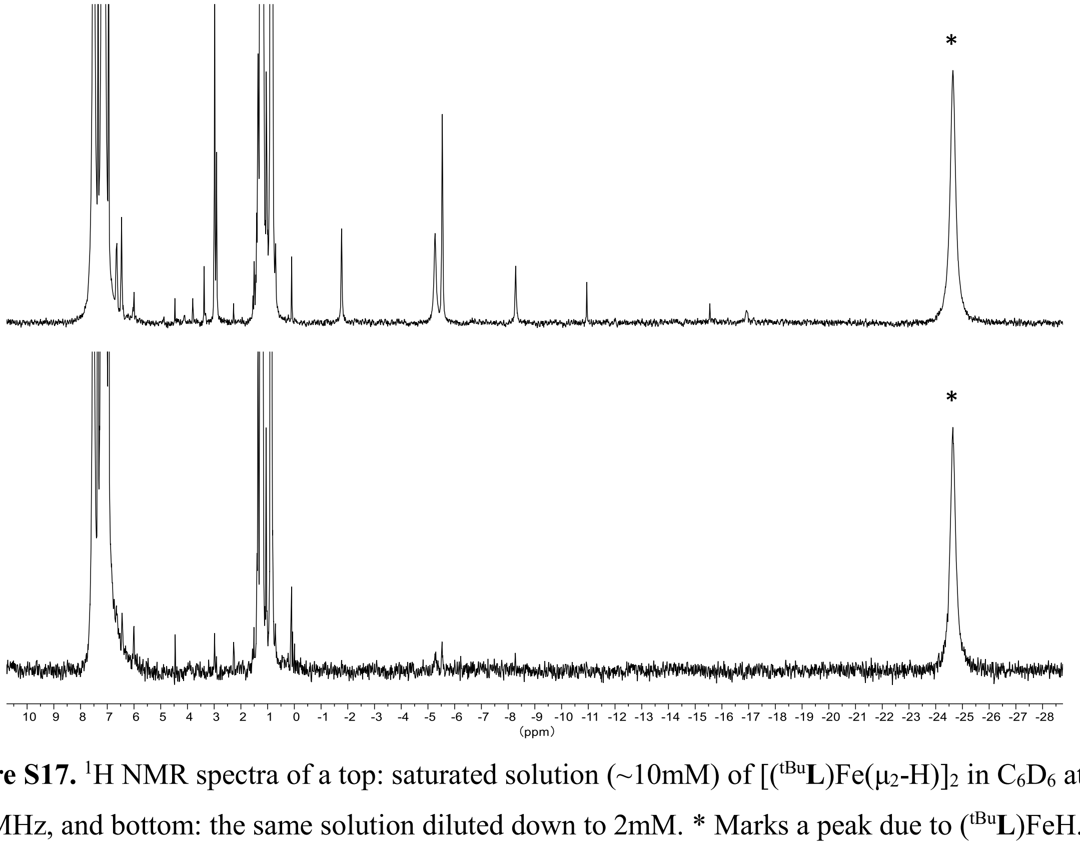

Figure S17. ^{1}H NMR spectra of a top: saturated solution (~10mM) of $[(^{tBu}\mathbf{L})Fe(\mu_2\text{-}H)]_2$ in C_6D_6 at 400 MHz, and bottom: the same solution diluted down to 2mM. * Marks a peak due to $(^{tBu}\mathbf{L})FeH$.

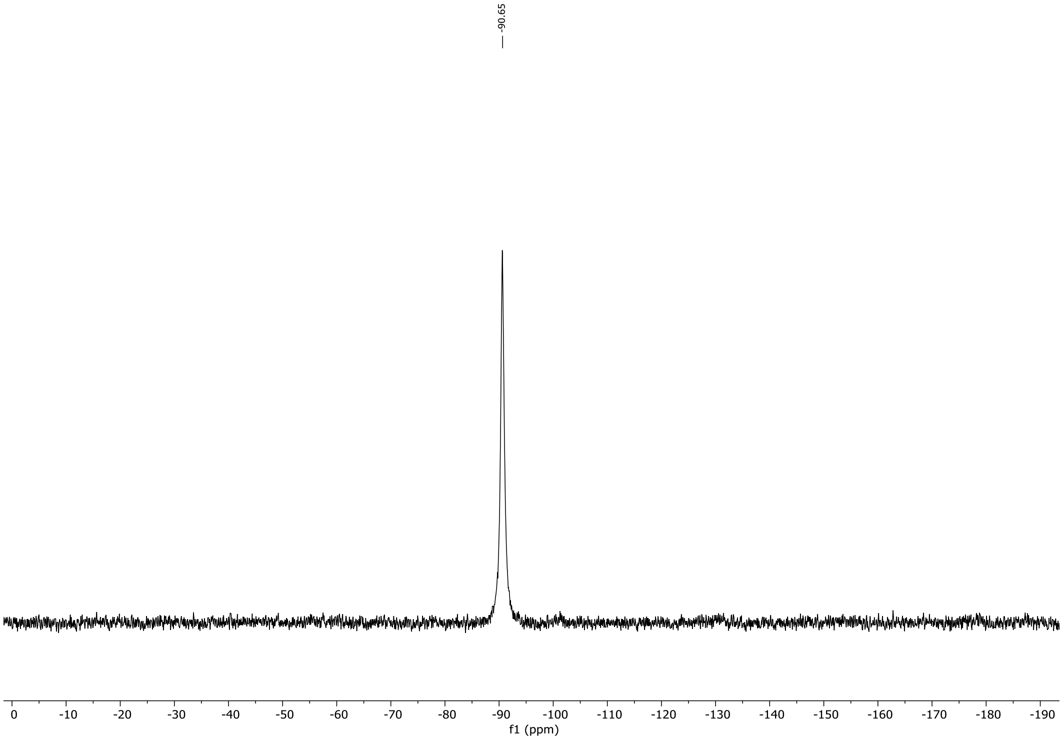

Figure S18. ^{19}F NMR spectrum of $[(^{tBu}\mathbf{L})Fe(\mu_2\text{-}H)]_2$ in C_6D_6 at 376 MHz.

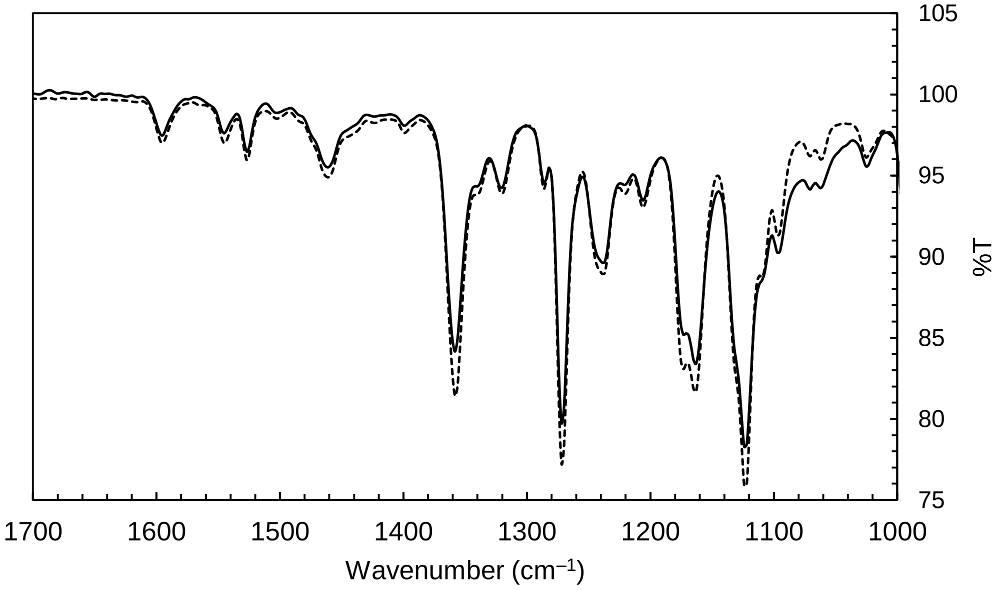

Figure S19. FTIR spectrum of $[(^{tBu}\mathbf{L})Fe(\mu_2\text{-}H)]_2$ (solid line) and $[(^{tBu}\mathbf{L})Fe(\mu_2\text{-}D)]_2$ (dashed line).

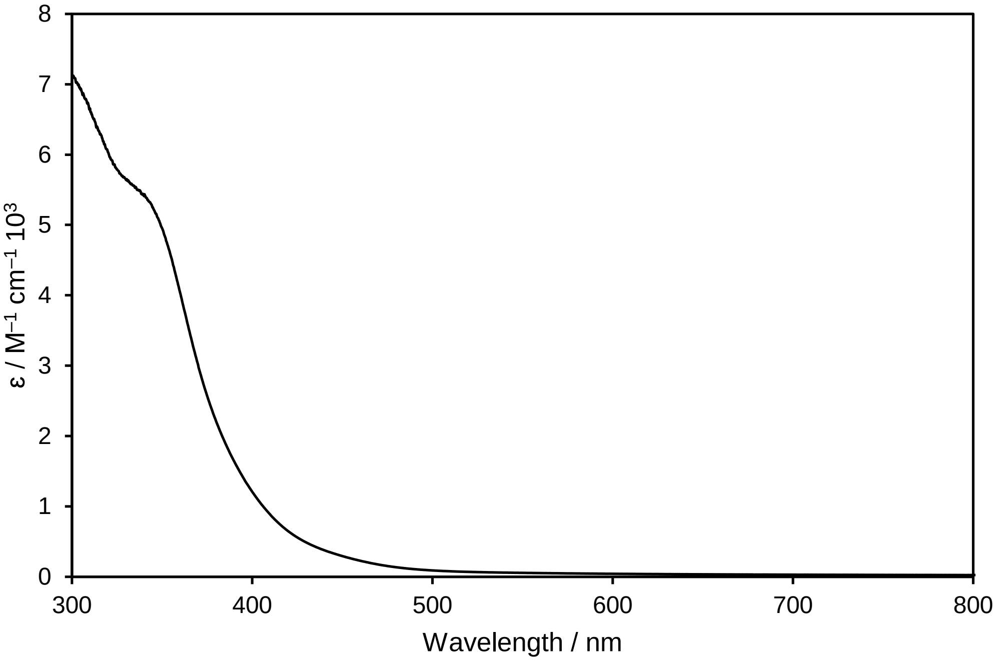

Figure S20. UV-Vis spectrum of $[(^{tBu}\mathbf{L})Fe(\mu_2\text{-}H)]_2$ in C_6H_6.

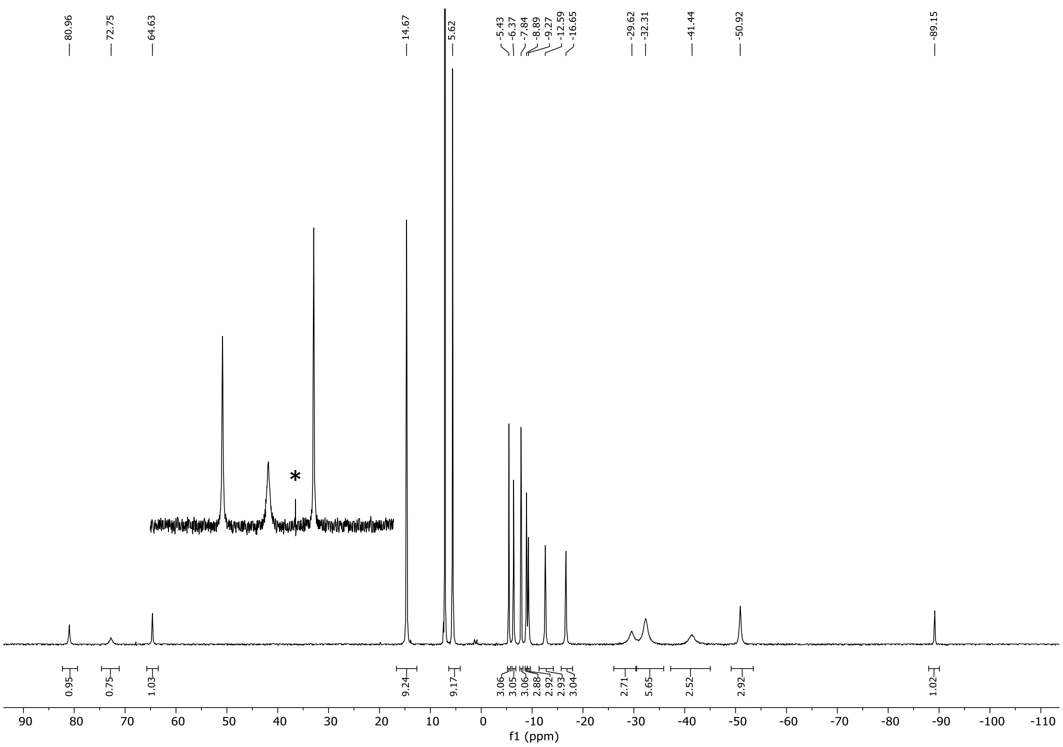

Figure S21. ^{1}H NMR spectrum of (^{Ad}**L**)FeI in C_6D_6 at 400 MHz. Downfield region, inset. * Marks an instrument artifact.

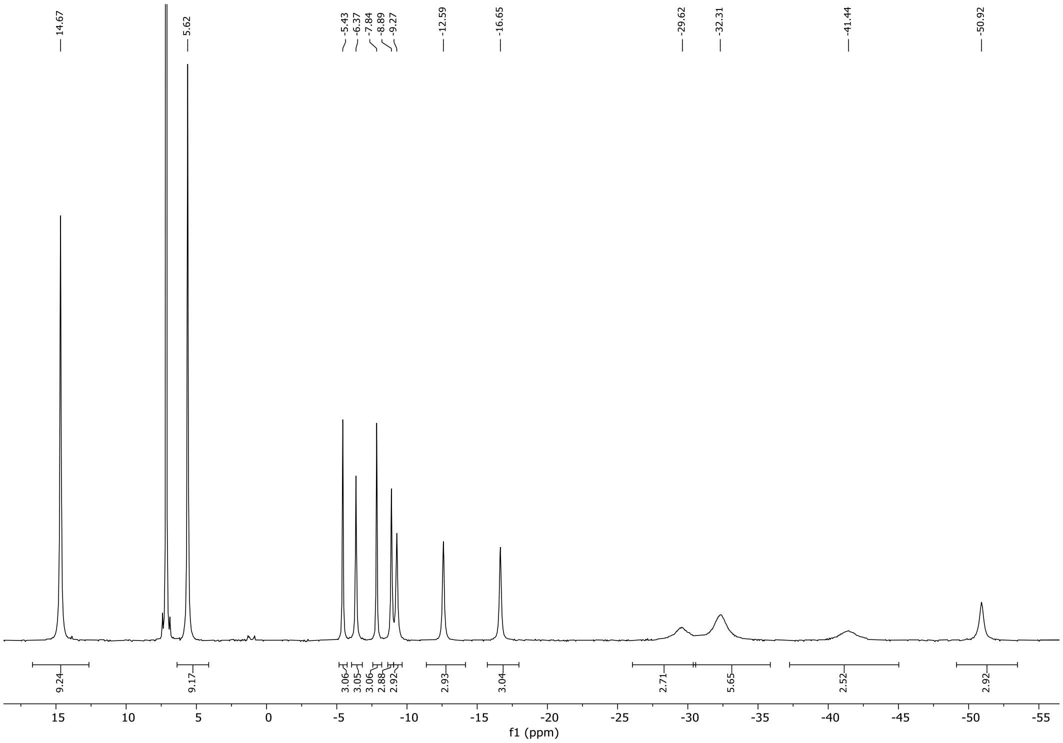

Figure S22. ^{1}H NMR spectrum of (^{Ad}**L**)FeI in C_6D_6 at 400 MHz, middle region.

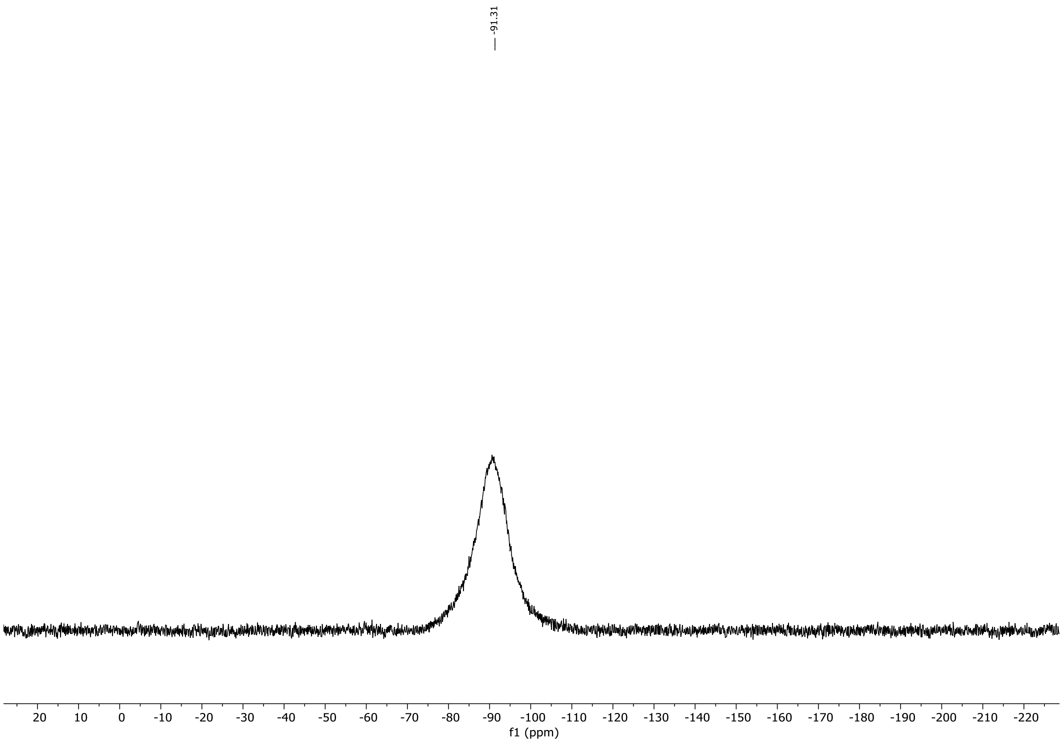

Figure S23. ^{19}F NMR spectrum of (^{Ad}**L**)FeI in C_6D_6 at 376 MHz.

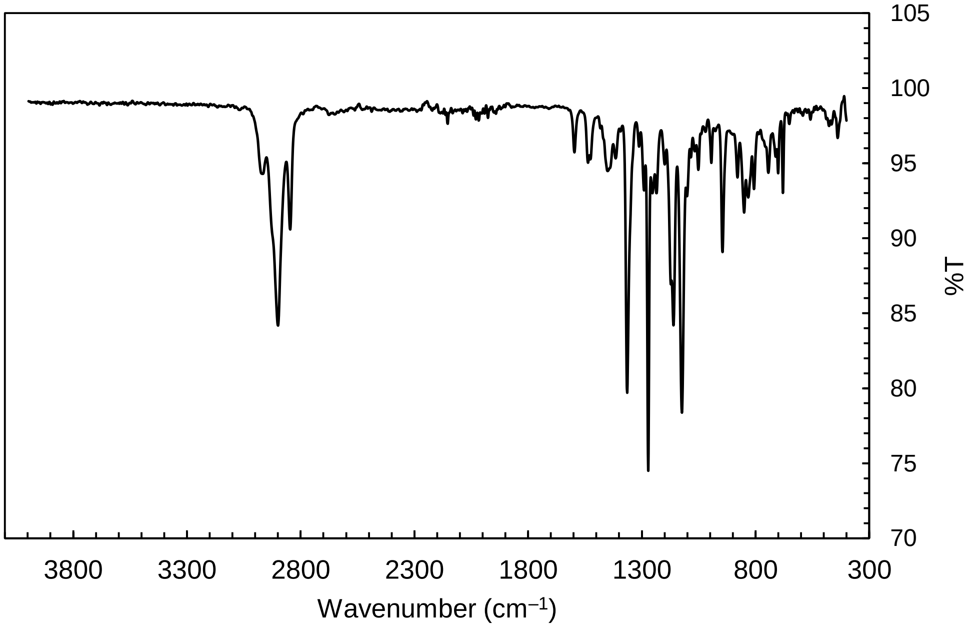

Figure S24. FTIR spectrum of (^{Ad}**L**)FeI.

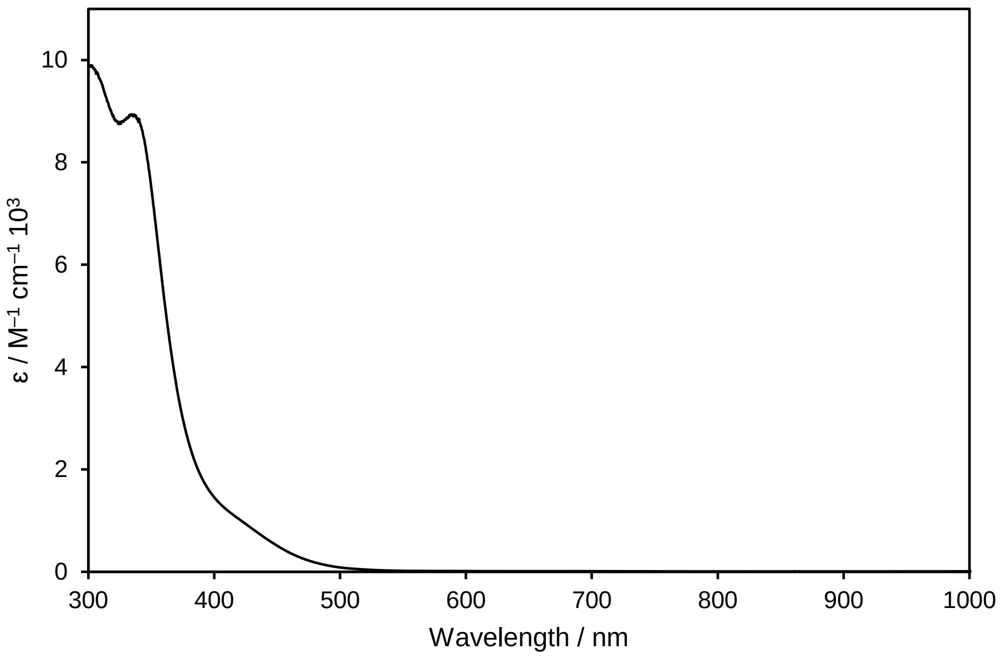

Figure S25. UV-Vis spectrum of (^{Ad}**L**)FeI in C_6H_6.

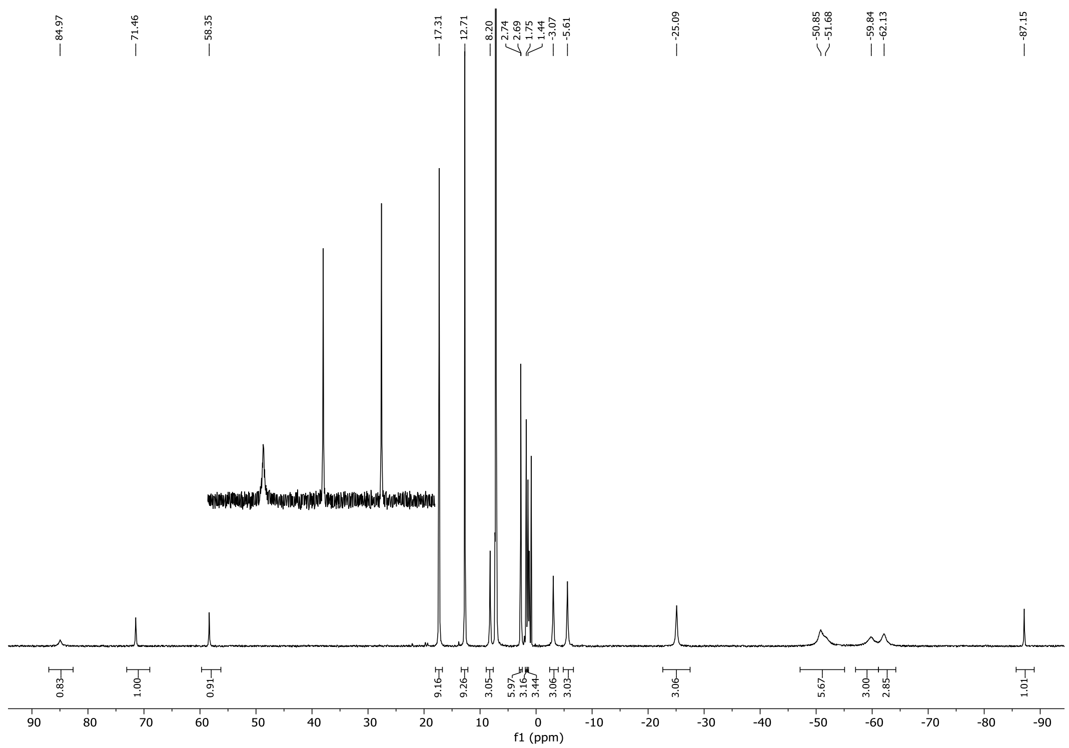

Figure S26. ^{1}H NMR spectrum of (^{Ad}**L**)FeH in C_6D_6 at 400 MHz. Downfield region, inset.

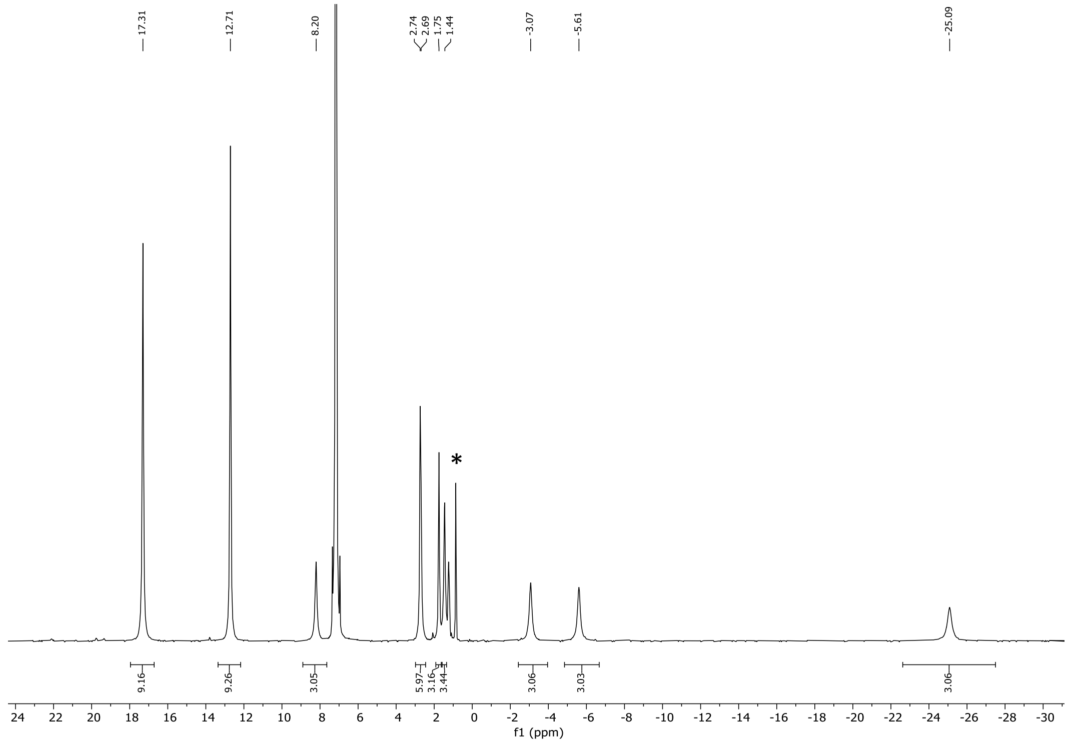

Figure S27. ^{1}H NMR spectrum of (^{Ad}**L**)FeH in C_6D_6 at 400 MHz, middle region. * Marks peaks due to n-pentane of crystallization.

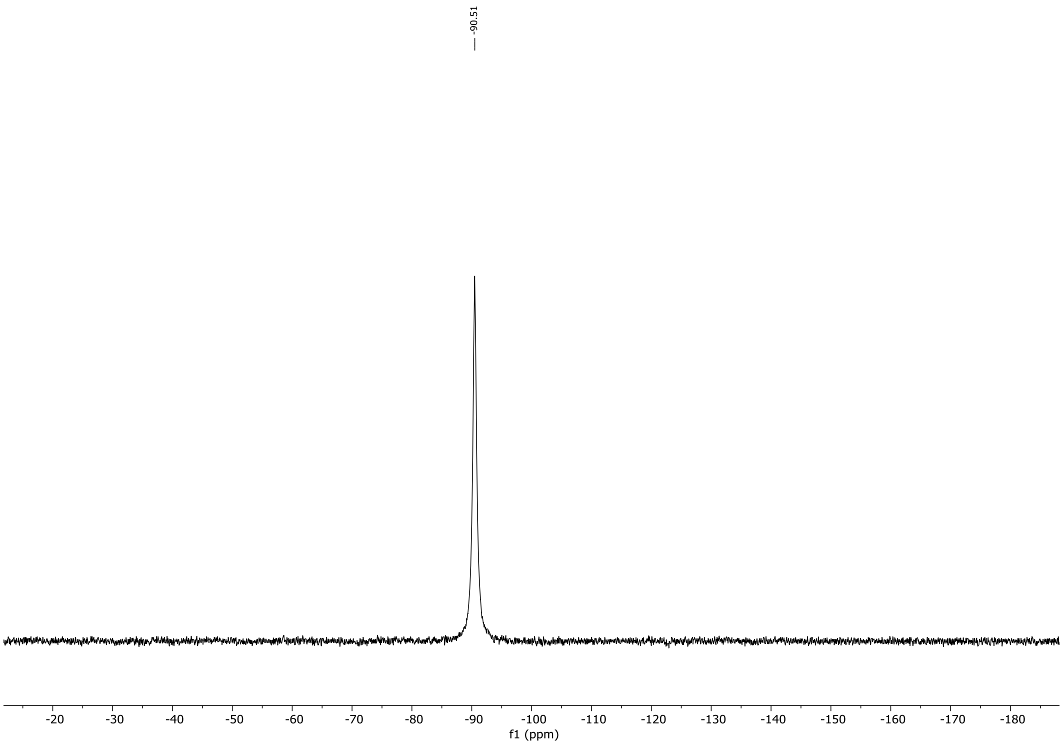

Figure S28. ^{19}F NMR spectrum of (^{Ad}**L**)FeH in C_6D_6 at 376 MHz.

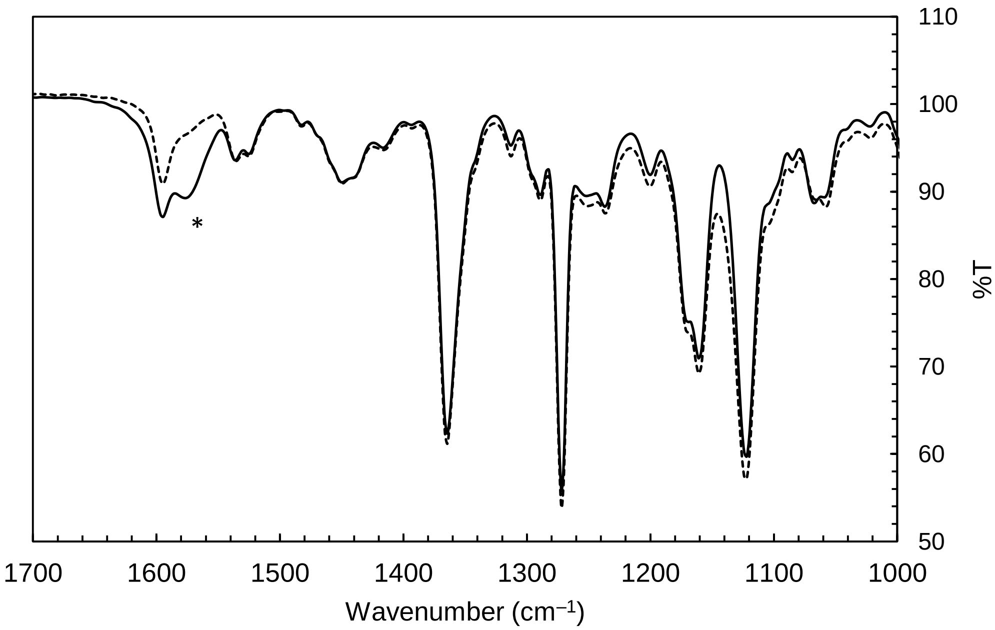

Figure S29. FTIR spectrum of (^{Ad}**L**)FeH (solid line) and (^{Ad}**L**)FeD (dashed line). * marks the Fe–H stretching band.

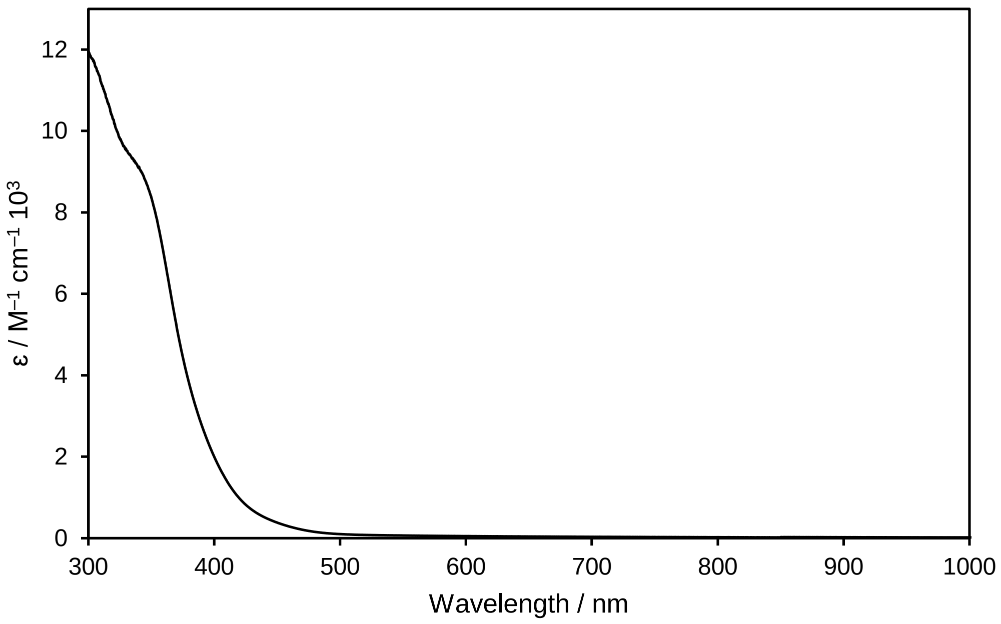

Figure S30. UV-Vis spectrum of (^{Ad}**L**)FeH in C_6H_6.

Additional Data

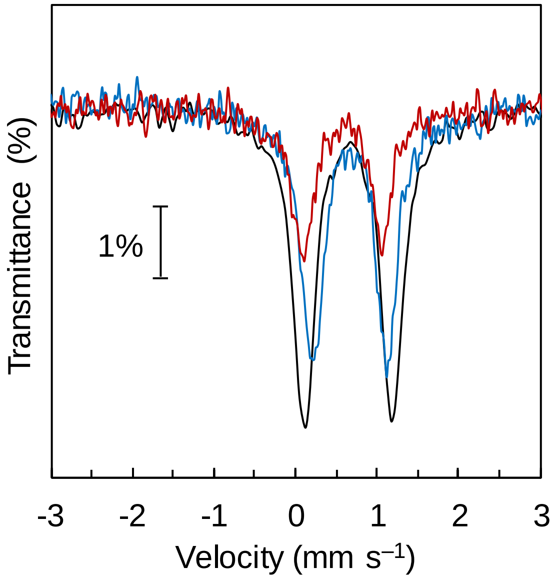

Figure S31. Temperature dependence of the Mössbauer spectrum of (^{Ad}**L**)FeH: 150 K (red trace); 80 K (black trace, reproduced from the main text) and 5 K (blue trace).

Table S1. Breakdown of calculated Fe core electron densities for different DFT functionals.

		BP86		TPPSh		B3LYP	
			%		%		%
	ρ_0^{1s}	10474.5011	90.45	10476.0055	90.45	10473.4543	90.43
	ρ_0^{2s}	967.9752	8.358	968.8700	8.365	969.9789	8.375
(***L**)FeI	ρ_0^{3s}	134.4741	1.161	133.6178	1.154	134.0917	1.158
	ρ_0^{val}	3.9592	0.03419	3.7292	0.03220	3.8328	0.03309
	ρ_0	11580.9096		11582.2224		11581.3578	
			%		%		%
	ρ_0^{1s}	10474.4793	90.44	10475.9826	90.44	10473.4331	90.43
	ρ_0^{2s}	968.0198	8.358	968.9110	8.365	970.0225	8.375
(***L**)FeH	ρ_0^{3s}	134.5854	1.162	133.7549	1.155	134.2151	1.159
	ρ_0^{val}	4.5279	0.03910	4.2487	0.03668	4.3368	0.03744
	ρ_0	11581.6125		11582.8972		11582.0075	
			%		%		%
	ρ_0^{1s}	10474.4911	90.44	10475.9911	90.44	10473.4444	90.43
	ρ_0^{2s}	968.0547	8.358	968.9429	8.365	970.0579	8.375
[(***L**)Fe(μ_2-H)]$_2$	ρ_0^{3s}	134.6187	1.162	133.8029	1.155	134.2613	1.159
	ρ_0^{val}	4.6482	0.04013	4.3221	0.03731	4.4404	0.03834
	ρ_0	11581.8127		11583.0590		11582.2041	
